# Transcriptional responses of *Arabidopsis thaliana* to chewing and sucking insect herbivores

**DOI:** 10.3389/fpls.2014.00565

**Published:** 2014-11-14

**Authors:** Heidi M. Appel, Howard Fescemyer, Juergen Ehlting, David Weston, Erin Rehrig, Trupti Joshi, Dong Xu, Joerg Bohlmann, Jack Schultz

**Affiliations:** ^1^Bond Life Sciences Center and Division of Plant Sciences, University of MissouriColumbia, MO, USA; ^2^Department of Biology, The Pennsylvania State UniversityUniversity Park, PA, USA; ^3^Michael Smith Laboratories, University of British ColumbiaVancouver, BC, Canada; ^4^Department of Biology, University of VictoriaVictoria, BC, Canada; ^5^Biosciences Division, Oak Ridge National LaboratoryOak Ridge, TN, USA; ^6^Biology and Chemistry Department, Fitchburg State UniversityFitchburg, MA, USA; ^7^Department of Computer Science, Bond Life Sciences Center, Informatics Institute, University of MissouriColumbia, MO, USA

**Keywords:** *Arabidopsis thaliana*, *Spodoptera exigua*, *Pieris brassicae*, *Myzus persicae*, *Brevicoryne brassicae*, herbivory, hormone signaling, glucosinolates

## Abstract

We tested the hypothesis that *Arabidopsis* can recognize and respond differentially to insect species at the transcriptional level using a genome wide microarray. Transcriptional reprogramming was characterized using co-expression analysis in damaged and undamaged leaves at two times in response to mechanical wounding and four insect species. In all, 2778 (10.6%) of annotated genes on the array were differentially expressed in at least one treatment. Responses differed mainly between aphid and caterpillar and sampling times. Responses to aphids and caterpillars shared only 10% of up-regulated and 8% of down-regulated genes. Responses to two caterpillars shared 21 and 12% of up- and down-regulated genes, whereas responses to the two aphids shared only 7 and 4% of up-regulated and down-regulated genes. Overlap in genes expressed between 6 and 24 h was 3–15%, and depended on the insect species. Responses in attacked and unattacked leaves differed at 6 h but converged by 24 h. Genes responding to the insects are also responsive to many stressors and included primary metabolism. Aphids down-regulated amino acid catabolism; caterpillars stimulated production of amino acids involved in glucosinolate synthesis. Co-expression analysis revealed 17 response networks. Transcription factors were a major portion of differentially expressed genes throughout and responsive genes shared most of the known or postulated binding sites. However, *cis*-element composition of genes down regulated by the aphid *M. persicae* was unique, as were those of genes down-regulated by caterpillars. As many as 20 *cis*-elements were over-represented in one or more treatments, including some from well-characterized classes and others as yet uncharacterized. We suggest that transcriptional changes elicited by wounding and insects are heavily influenced by transcription factors and involve both enrichment of a common set of *cis*-elements and a unique enrichment of a few *cis*-elements in responding genes.

## Introduction

Approximately a quarter of all described eukaryotic species are insects that feed on plants, and as a group they are thought to exert strong selection on plants to detect and repel them (Ehrlich and Raven, 1964; Futuyma and Agrawal, 2009). Any individual plant may be attacked by many species of herbivores feeding in a variety of ways. Some species feed by chewing tissues, which involves wounding and water loss, while others feed by inserting needle-like mouthparts (stylets) between and into plant cells, causing less overall damage. Some vector plant diseases whereas others inadvertently introduce microbial pathogens from leaf surfaces or gut contents. Although field studies of plant evolution in response to herbivory are rare, they indicate substantial selection for plant resistance traits (Agrawal et al., 2012; Zust et al., 2012).

Many potential plant resistance traits are modified or enhanced in response to insect attack and can comprise a significant barrier to insect feeding and concomitant pathogen introduction (Karban and Baldwin, 1997; Dicke and Hilker, 2003). Since insects wound as they feed, there is always a question of whether these changes are general responses to wounding or are specific to the insect species. Although there are currently few examples of gene-for-gene recognition systems in plant-herbivore interactions analogous to pathogen effector triggered immunity (Rossi et al., 1998; Aggarwal et al., 2014), specificity in plant phenotypic responses to different herbivores is commonplace (Ali and Agrawal, 2012; Barrett and Heil, 2012). Not surprisingly, responses to sucking insects usually differ from those elicited by chewing insects. More surprising is that biochemical responses can be herbivore species-specific, such that some insects suppress or fail to elicit defense responses (Alba et al., 2012). Although the basis of species-specific responses is largely unknown, the different responses elicited by insects and mechanical wounding support the view that elicitors in saliva or regurgitant cue them (Bonaventure et al., 2011; Maffei et al., 2012). Based on the pathogen recognition system of plants, a model of insect recognition by plants has been proposed with herbivore-associated molecular patterns (HAMPs) and damage-associated molecular patterns (DAMPs) cueing downstream HAMPs-triggered immunity (HTI) (Heil, 2009).

Since transcriptional reprogramming underlies defense responses, HAMPs should reflect underlying transcriptional changes. There are now many published studies of transcriptional change in plants responding to insect herbivores (Heidel-Fischer et al., 2014). Drawing conclusions among them is confounded by the many differences in experimental approaches, including herbivore species and treatments, plant species and tissues, sampling times, and gene expression platforms. Replicated, whole-genome expression profiling is important to comparative studies because it can reveal the full range of transcriptional responses, unlike partial genome arrays or targeted quantitative reverse-transcription polymerase chain reaction (qRT-PCR). The age of plant tissue treated and sampled should be consistent to address the large effect that age can have on gene expression profiles. Use of multiple herbivores and sampling times is also important for comparative studies because plant responses to different herbivores can differ qualitatively and quantitatively over time.

To identify HAMPs that address these experimental issues, we report here a fully replicated study of genome-wide transcriptional responses by *Arabidopsis thaliana* (L.) to wounding, to chewing insects (caterpillars) and to stylet feeders (aphids). We used a genome-wide *Arabidopsis* microarray to examine all transcriptional changes at two time intervals after mechanical wounding or being attacked by four insect species. The insect species were two aphids (phloem feeders) and two caterpillars (leaf chewers). Both feeding types included one with an extremely broad diet (generalist) and another with a narrow diet (specialist) focused on Brassicaceae, the plant family to which *A. thaliana* belongs. This design enabled testing the hypothesis that *Arabidopsis* can recognize and respond individually to these insects at the transcriptional level. Common patterns in fundamental reprogramming of plant metabolism were identified using gene enrichment tests. Candidate genes that may determine species-specific responses were identified with coexpression network analysis. Frequencies determined for coregulated gene promoters linked to genes differentially expressed suggest that reprogramming of expression patterns elicited by wounding and different insect species involve both a common set and a unique, small set of *cis*-elements.

## Methods

### Insects and plants

We assessed the transcriptional responses in rosette leaves of 4-week-old *A. thaliana* ecotype Columbia Wild-Type (Col WT) to attack by larvae of two leaf chewing caterpillar species and by adults and nymphs of two phloem feeding aphid species (Table 1). The caterpillars were *Spodoptera exigua* (Hübner) (Noctuidae), which feeds on as many as 20 plant families including Brassicaceae (Greenberg et al., 2001), and *Pieris rapae* (L.) (Pieridae), which feeds exclusively on plants in the family Brassicaceae, to which *Arabidopsis* belongs (Renwick and Lopez, 1999). The aphids (both in Aphididae) were *Myzus persicae* (Sulzer), a broad generalist feeding on species in many plant families including Brassicaceae, and *Brevicoryne brassicae* (L.), whose feeding is limited to the Brassicaceae (Blackman and Eastop, 1994). We henceforth refer to *S. exigua* and *M. persicae* as “dietary generalists” and *P. rapae* and *B. brassicae* as “dietary specialists.” Both aphids were maintained as plant virus free clones on pak-choi plants (*Brassica campestris* L. ssp. *chinensis* cv. Black Behi). Eggs of *S. exigua* were obtained from Benzon Research and larvae were reared on artificial diet (Bio-Serv). *Pieris rapae* was maintained as a culture in our lab on pak-choi and originated from the Carolina Biological Supply Company. Both caterpillar species were transferred to Col WT plants one day before the experiments for acclimation to the new host. Plant seeds were vernalized in 2% agar and sown into 6 × 5 cm pots containing sterile Metromix 200 soil (Sun Gro Horticulture). Plants were chamber grown at 22 ± 1°C, 65 ± 5% relative humidity, and 200 μmol m^−2^s^−1^ light intensity on a 8:16 (L:D) photoperiod. Plants were watered as needed and fertilized every other watering with 21-7-7 Miracle-Gro (Scotts Company).

**Table 1:** **Experimental design and key to treatments**.

**Abbreviation**	**Insect**	**Leaf Type**	**Time (h)**	**N**
Pr-L-6 h	*Pieris rapae*	Local = Attacked	6	3
Pr-L-24 h	*Pieris* rapae	Local = Attacked	24	4
Pr-S-6 h	*Pieris rapae*	Systemic = Unattacked	6	4
Pr-S-24 h	*Pieris rapae*	Systemic = Unattacked	24	4
Se-L-6 h	*Spodoptera exigua*	Local = Attacked	6	3
Se-L-24 h	*Spodoptera exigua*	Local = Attacked	24	4
Se-S-6 h	*Spodoptera exigua*	Systemic = Unattacked	6	4
Se-S-24 h	*Spodoptera exigua*	Systemic = Unattacked	24	4
Wo-L-6 h	Wounding	Local = Attacked	6	3
Wo-L-24 h	Wounding	Local = Attacked	24	4
Wo-S-6 h	Wounding	Systemic = Unattacked	6	3
Wo-S-24 h	Wounding	Systemic = Unattacked	24	4
Bb-6 h	*Brevicoryne brassicae*	Local + Systemic	6	4
Bb-24 h	*Brevicoryne brassicae*	Local + Systemic	24	4
Mp-6 h	*Myzus persicae*	Local + Systemic	6	4
Mp-24 h	*Myzus persicae*	Local + Systemic	24	4

### Experimental plant treatments

The treatments are summarized in Table 1. The caterpillar treatment was designed to capture early gene expression events and minimize variation due to leaf age and amount of insect damage. All leaves selected for treatment and harvest were fully expanded mature leaves. Second and third instar (*N* = 6–10) caterpillars of both species were allowed to feed for 2–4 h to generate 6 leaves of similar age per plant with ~20% leaf area removed. Caterpillars were wrangled as needed with a size 0 camel's hair brush to concentrate their feeding on the 6 leaves sampled. Caterpillars were removed when sufficient damage was achieved and the plants were returned to the growth chamber. The mechanical wounding treatment was designed to approximate insect damage to tissues by running a sterile pattern wheel across both sides of the midrib of 6 leaves of similar age on each plant, once at the beginning of the caterpillar treatment and again half way through the caterpillar wrangling period. Control plants were jiggled with the brush to simulate leaf movement caused by caterpillar wrangling or mechanical wounding. Damaged leaves (“local” to the attack) were harvested for gene expression 6 and 24 h after the start of caterpillar damage or wounding. Unwounded leaves (“systemic”) were harvested separately from size-matched damaged or wounded leaves. Leaves from 3 to 4 plants per treatment (caterpillar, wounding, and controls) were pooled for each of the four biological replicates.

Treatment of plants with aphids was different from that for caterpillars because aphids have effects on plants that are much weaker and slower to develop than those of caterpillars (Mewis et al., 2005, 2006) and aphids cannot be readily contained on individual leaves. Sub-adult (final instar) and adult aphids (*N* = 20) were placed on plants whose rosettes were caged at the soil line by transparent mylar cylinders (5 cm diameter, 9 cm high) with tops of fine mesh gauze (<0.01 mm mesh wide) to maintain air exchange. Controls were caged plants without aphids and all plants were returned to the growth chamber. After 1 week, all cages and aphids were removed and control plants were jiggled with a camel's hair brush to simulate leaf movement caused by aphid removal. Plants were returned to the growth chamber and whole plants were harvested per treatment (aphid and control) for gene expression 6 and 24 h after aphid removal.

### RNA isolation

Total RNA was isolated from leaves using a modified TRIZOL extraction method as follows. Approximately 0.5 g of plant material was ground to a powder in liquid nitrogen using mortar and pestle, resuspended without thawing in 6 ml TRIZOL reagent (Invitrogen) by vortexing, and incubated at 65°C for 5 min with regular mixing. Cell debris was pelleted by centrifugation (30 min, 12,000 g, 4°C) and the supernatant was extracted twice with 3 ml chloroform with the aqueous phase recovered each time after centrifugation (20 min, 12,000 × g, 4°C). RNA was precipitated from this phase at room temperature for 5 min with 0.5 volumes each of 0.8 M sodium citrate and isopropanol. The RNA pellet obtained after centrifugation (30 min, 12,000 × g, 4°C) was washed with 70% ethanol, recovered again by centrifugation, air dried for 5 min and resuspended in 200 μl nuclease free water. The RNA was further purified by standard ethanolic sodium acetate precipitation at −20°C overnight. Following a wash with 70% ethanol the pelleted RNA (30 min, 12,000 g, 4°C) was air dried and resuspended in nuclease free water to an approximate concentration of 5 μg/μl whose actual concentration was determined spectrophotometrically. Quality of randomly selected samples of RNA was determined using a 2100 Bioanalyzer (Agilent Technologies).

### Microarrays and the preparation and hybridization of cDNA to arrays

Design and production of microarrays with 26,090 *Arabidopsis* oligonucleotide targets (Qiagen Operon), 12 housekeeping gene oligos (Qiagen Operon) as positive controls and 16 oligos with no similarity to any *Arabidopsis* gene (4 synthesized human genes and 12 others) as negative controls and internal, spike controls was previously described (Ehlting et al., 2005). Location of all oligos and orientation marker was also previously described (Ehlting et al., 2005) and provided in the platform file deposited to the NCBI GEO database (http://www.ncbi.nlm.nih.gov/geo/; series accession GSE62287).

Total RNA isolated as previously described (Ehlting et al., 2005) was used for a direct labeling procedure that generated the cDNA hybridized microarray slides. All procedures for labeling, microarray hybridization, image scanning, and the identification and quantification of spots were performed as previously described (Ehlting et al., 2005, 2008). Microarray experiments involved hybridizing three or four replicate arrays per treatment. The RNA from control plants harvested with each treatment was pooled within time points to obtain sufficient control RNA. Labeled cDNA derived from this control RNA was co-hybridized with labeled cDNA derived from RNA isolated from independent biological replicates receiving insects or mechanical wounding, resulting in four biological replicates per treatment. Dye bias was accounted for by swapping dye labeling between treatment and control samples among bioreplicates within each treatment group. Hybridization order was randomized to avoid biases due to the hybridization time.

### Microarray data analysis

Analyses of gene specific elements used customized scripts for R and Bioconductor (Team, 2014). Background correction performed as previously described (Ehlting et al., 2008) excluded on average 19% (12–27%) of all spots from further analyses as non-detectable. Background corrected signal intensities were used for subsequent Loess normalization and statistical testing (Student's *t*-test, analysis of variance, false discovery rate of expression ratios) between treatment and corresponding control, as previously described (Ehlting et al., 2008). The expression data was initially filtered to obtain genes with a *t*-test *p* < 0.05 and fold-change >3 between treatment and control in at least one sample. Normalized expression ratios for these genes from all treatments were then used to perform an analysis of variance (ANOVA) and an estimate of the false discovery rate (FDR) based on the distribution of parametric *p*-values (Supplemental Figure 1). Means of the normalized expression ratios were subjected to a hierarchical clustering analysis with average linkage using Genesis v1.2 (Institute for Biomedical Engineering).

A high estimated FDR arose from our experimental design intended to capture the biological variation inherent in plant herbivore interactions (biological replicates treated with a small number of herbivores that behaved individually). Lower *p*-values from *t*-tests were associated with a lower false discovery rate (Supplemental Table 1). However, a relatively large number of array probes were associated with high *p*-values which still contain a substantial number of truly differentially expressed genes as estimated from the higher frequency of genes in these *p*-value bins compared to the frequency expected if no genes were differentially expressed (indicated by the horizontal line in Supplemental Figure 1). Although a *p*-value cut-off of 0.01 would reduce the number of falsely discovered genes, a substantial number of truly differentially expressed genes would also be missed. Therefore, we assumed that high fold change difference is associated with a lower likelihood of being a false positive (Pylatuik and Fobert, 2005) to initially obtain 3123 genes as “differentially expressed” (i.e., genes with treatment-induced change in transcript abundance) as those genes for each time point that were associated with a *t*-test *p* < 0.05 (accepting a false discovery rate of up to 0.3) and also displayed a more than two-fold change between treatment and control (Supplemental Table 1). After removing genes identified merely as chromosome loci, BAC clones, and other annotations not clearly known to produce functional proteins, 2778 genes remained in the differentially expressed category (Supplemental Table 2). All further analyses were done on this set of genes.

### Targeted evaluation of microarray expression data

To compare statistically significant patterns of gene expression from the array with those from qPCR, we measured qPCR expression of AP2-ERF transcription factors (*N* = 17) in the caterpillar treatments at 6 and 24 h in both local and systemic tissue because of their species-specific expression pattern, (Rehrig et al., 2011) for a total of 136 measurements. There were many cases (36%) in which the more sensitive qPCR detected statistically significant changes in expression not detected by the array. However, a majority of those identified by the array as significant were confirmed by qPCR as significant (20 of 26). Four of the 6 array false positives had qPCR values for relative expression in the same direction as those of the array even though they failed the test of significance, a more stringent criterion than most authors apply (Rehrig et al., 2011).

### Clustering and overlap analyses

The overall pattern of similarity and difference in gene expression among the treatments was identified with a simple clustering algorithm (VARCLUS, SAS) that used the centroid method and the maximum number of possible clusters set to 16, or the number of treatments. The percentage overlap in gene expression between treatments was calculated as the (# genes in common/(sum # genes elicited by both treatments–# genes in common).

To identify functional patterns in the large number of genes differentially expressed in response to insects, we used the DAVID gene enrichment analysis tool (Huang et al., 2008). The gene lists for each treatment consisted of only those that were statistically up or down regulated in that treatment compared to controls (Supplemental Table 3) and the classification stringency was set to medium. Functional clusters were identified based on the modified Fisher exact *p*-value (EASE score) and clusters with a Group Enrichment Score > 1 were examined further. A Benjamini-Hochberg *p* < 0.05 correcting for multiple comparisons was considered to be statistically enriched; values significant at the uncorrected *p*-value were also indicated in some comparisons. Similar analyses were run using MAPMAN and PAGEMAN but no significant enrichments were detected with a Benjamin-Hochberg correction for multiple comparisons. Thus, we concluded that the large number of genes of unknown function in this study made DAVID a more useful tool because it incorporates a larger number of databases.

The Hormonometer Tool (Volodarsky et al., 2009) was used to evaluate the similarity in expression profiles elicited by insects with the published, indexed list of those elicited by exogenous application of plant hormones. Although exogenously applied hormones may not represent *in vivo* levels of hormones, this kind of comparison is frequently used to identify the importance of hormone signaling pathways in plant responses. *Arabidopsis* gene identities (AGI) were converted to Affymetrix GeneChip identities using the “at to AGI converter” tool (The Bio-Analytic Resource for Plant Biology, http://bar.utoronto.ca/) (Supplemental Table 4). In a few cases there was no correspondence and the AGI data were omitted, while in a few other cases there were two GeneChip IDs for one AGI and the lines were duplicated and retained.

Groups of genes that are coregulated in response to treatments were identified with a weighted gene coexpression network analysis (WGCNA) as described previously (Zhang and Horvath, 2005; Weston et al., 2008, 2011). Background corrected signal intensities underwent variance stabilization and normalization followed by log_2_ transformation before entering network construction (Huber et al., 2002). The WGCNA analysis consisted of 4 steps: (1) creation of a pair-wise Pearson correlation matrix for all genes across all treatments; (2) transformation of correlations to connection strengths (connectivity) using a signed power adjacency function (Zhu-Salzman et al., 2004); (3) identification of modules, or groups of highly correlated gene expression patterns by coupling hierarchical clustering with topological overlap matrix; and (4) relating external gene or treatment information to network properties. Correlations were corroborated using a random seed permutation *t*-test with 106 iterations.

The coexpression landscape of genes in functionally enriched categories in DAVID analysis was depicted with comparison to the ATTED-II database whose software generates networks by coexpressed ranks calculated from the 1388 GeneChip data (Obayashi and Kinoshita, 2010). AGI codes for genes of interest were pasted into the Network Drawer and the default settings were used to depict the network.

### Identification of promoter regions

Several on-line databases and bioinformatics tools were used to conduct three separate analyses to identify potential *cis*-elements involved in the signaling pathways after insect attack. Gene sequences up to 1000 bp upstream of the AGI transcription start site were downloaded from The *Arabidopsis* Information Network (TAIR; www.arabidopsis.org) Sequence Database for all genes whose expression was significantly affected by insect feeding or mechanical wounding. First, the database of motifs found in plant *cis*-acting regulatory DNA elements (PLACE; Higo et al., 1999) was used to search the downloaded sequences for all known transcription factor binding sites and *cis*-elements in known gene promoters. A customized Perl script was used to tally all elements found in each differentially regulated gene. Principal component analysis (PCA) and cluster analysis was performed with the tallied data to identify specific *cis*-element fingerprints possibly unique to insect treatments. Treatments were clustered on the basis of similarities of their *cis*-element distributions using the SAS VARCLUS procedure (SAS Institute).

Second, the ATHENA search tool (O'Connor et al., 2005), which uses a library of 105 known *Arabidopsis* transcription factor binding sites and 30,067 predicted promoters, was used to search the downloaded sequences for enriched, known transcription factor binding sites and *cis*-elements in gene promoters. The ATHENA algorithm conducts a student's *t*-test to determine whether motifs in a given sample set are significantly different from a random distribution in the genome. Motif occurrences with a *p* < 0.00001 were designated as “enriched.”

Third, MotifSampler (Thijs et al., 2001, 2002) was used to identify all potential (known and putative) transcription factor (TF) binding sites and enriched sequence consensus within the promoter regions in the downloaded, upstream sequences of differentially regulated genes. This tool successfully identified potential TF binding sites in a number of different species (Singh, 1998; Chen et al., 2002). Due to high nucleotide substitution rates, we wrote a short PHP script to place all combinations of A, C, G, or T's in place of degenerative nucleotide indicators (e.g., K, S, N, V). Resolved sequences were then compared against the ATHENA results.

## Results

### Transcriptional profiles were distinct, even within feeding types

**Figure 1:**
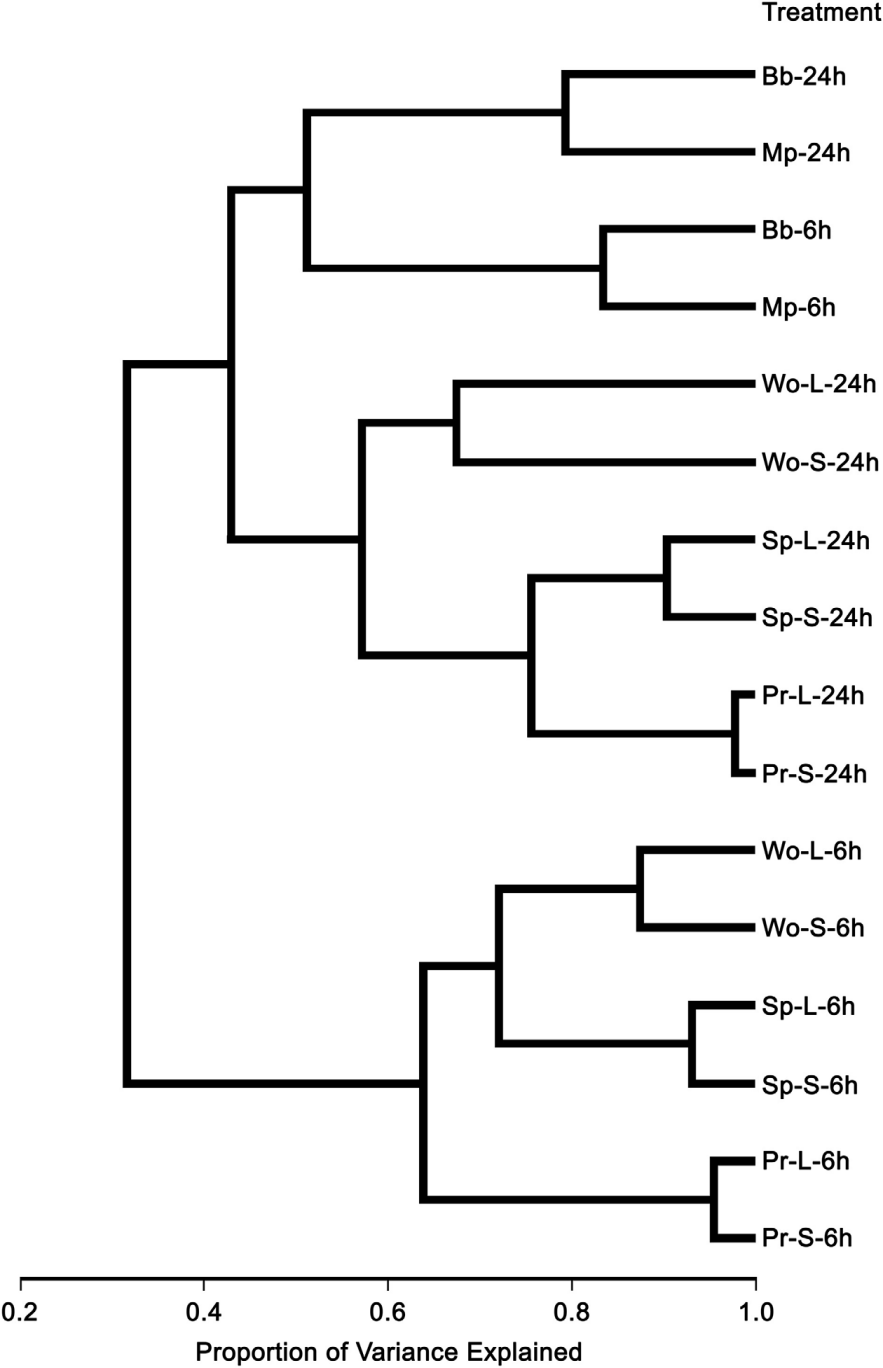
**Hierarchical cluster tree of *A. thaliana* genes differentially expressed in response to insect feeding and mechanical wounding**. Abbreviations for treatment names are the same as those used in Table 1.

We assessed the transcriptional responses in rosette leaves of 4-week-old *A. thaliana* Col plants to artificial wounding, attack by larvae of two species of leaf chewing caterpillar species and by adults and nymphs of two phloem feeding aphid species (Table 1). In all, 2778 genes on the full genome wide array were differentially expressed in one or more of our treatments, representing approximately 10.6% of the genes on the array having AGI annotation (Supplemental Table 2). Hierarchical clustering based on similarities (correlations) among the resulting transcriptional profiles (Figure 1) revealed three major clusters, one containing all aphid treatments, a second comprising wounding plus caterpillar treatments at 24 h, and the third comprising wounding plus caterpillar treatments at 6 h. The aphid cluster contained two subclusters based on sampling time. The wounding plus caterpillar cluster divided into two subclusters, one for wounding and another for the two caterpillar species; the latter contained sub subclusters for each species. The third cluster, responses to wounding and caterpillars at 6 h, divided into two subclusters, one for wounding and another for the insects, with the insect sub subcluster further divided by species. Hence feeding type (sucking vs. chewing) and sampling time (6 and 24 h) contributed most (about 40–60% of variance explained) to differentiating the transcriptional response profiles, whereas species contributed some (about 20% of variance explained) to differentiation and tissue treatment (wounded or not) contributed least (<10% of the variance) (Figure 1).

**Figure 2:**
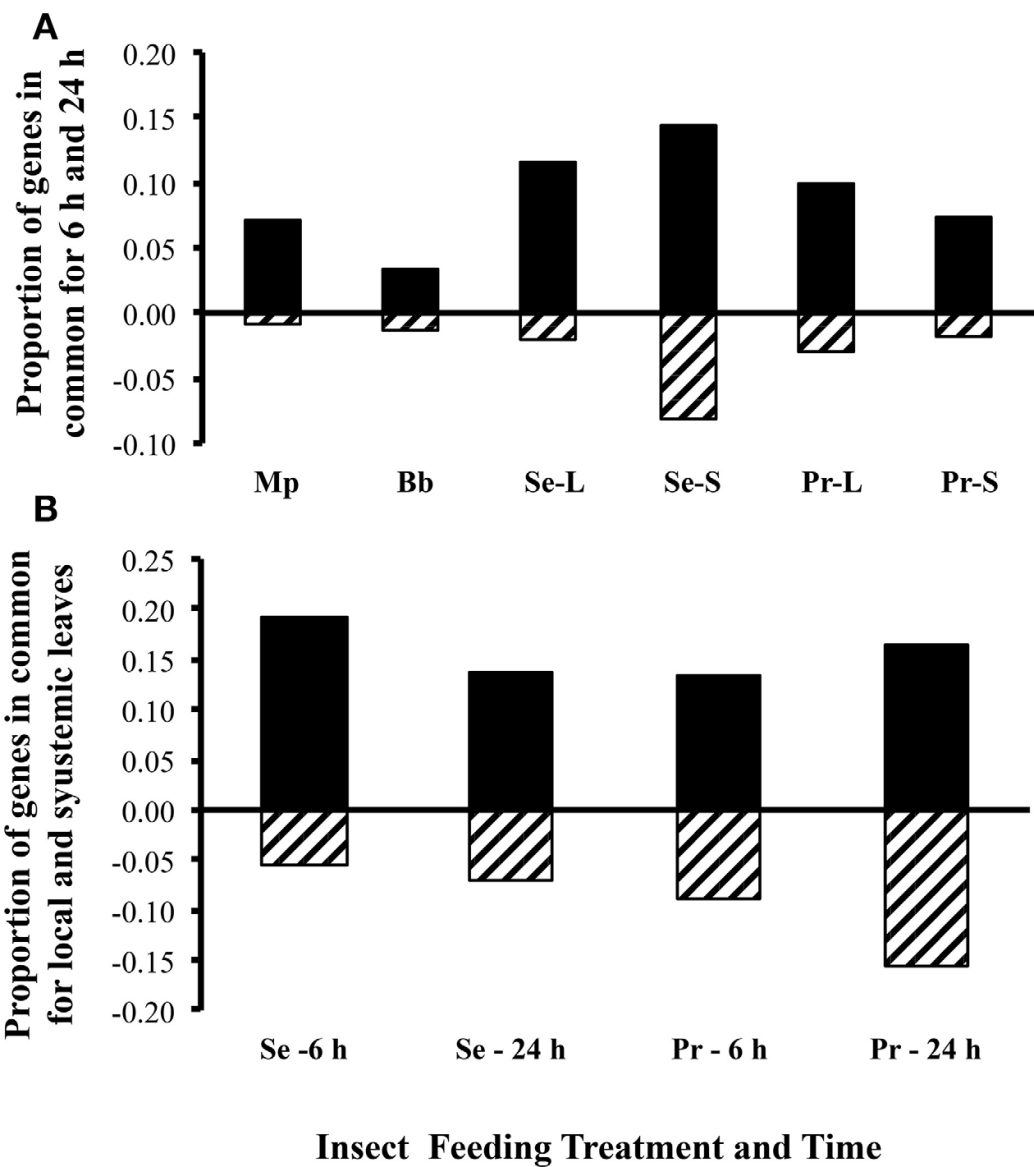
**Proportion of *A. thaliana* genes differentially expressed at both time points and leaf types**. **(A)** Proportion of genes differentially expressed at both time points, by treatment. **(B)** Proportion of genes differentially expressed in both leaf types, by treatment and time. Black bars, upregulated genes; cross hatched bars, downregulated genes; Mp, *Myzus persicae*; Bb, *Brevicoryne brassicae*; Se, *Spodoptera exigua*; Pr, *Pieris rapae*; L, local damaged leaves; S, systemic undamaged leaves (local and systemic leaves were from the same plant).

**Figure 3:**
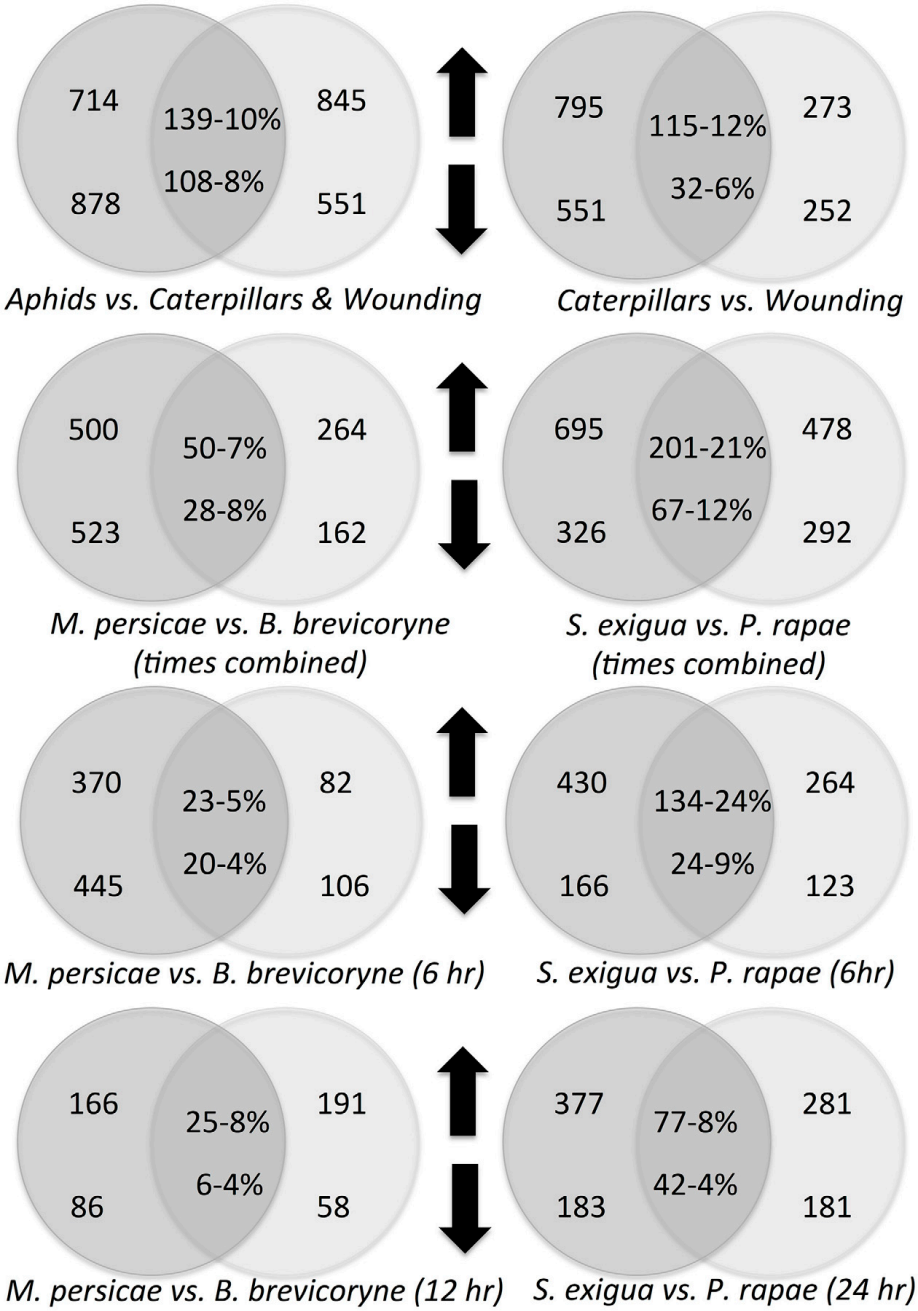
**Treatment comparisons with the number of genes differentially expressed in each treatment, the number of genes expressed in both treatments, and their proportional overlap**. Upward pointing arrows indicate upregulated genes, downward pointing arrows indicate downregulated genes.

*Arabidopsis* responses to treatments were highly dynamic (Figure 2). Since sampling was done 6 and 24 h after the removal of insects, the differences in expression between insects and control plants presumably represents a combination of induction and relaxation of gene expression responses. Across all treatments and tissues, overlap in genes differentially expressed at 6 and 24 h ranged from 3 to 15% and 1 to 8% for upregulated and downregulated genes, respectively (Figure 2A). The aphid *M. persicae* elicited far more differentially expressed genes at 6 h (*n* = 815, 45% up and 54% down) than at 24 h (*n* = 222, 74% up and 38% down) (Figure 3). In contrast, the aphid *B. brassicae* elicited fewer differentially expressed genes at 6 h (*n* = 188, 44% up and 56% down) than at 24 h (*n* = 249, 76% up and 23% down) Transcriptional responses to the two aphids across both times only shared a 4–8% overlap in differentially expressed genes. Both caterpillars elicited similar numbers of differentially expressed genes at 6 h and 24 h (Figures 2A, 3). However, the degree of overlap in these genes between the caterpillars differed with time, 24% up vs. 9% down at 6 h and 8% up vs. 4% down at 24 h for *S. exigua* and *P. rapae*, respectively. Feeding by *S. exigua* elicited the longest-lasting changes in gene expression and hence the most overlap in genes differentially expressed at both time points.

Response profiles differed between feeding type treatments. Aphids clustered with aphids and caterpillars with caterpillars plus wounding (Figure 1). Chewing caterpillars and sucking aphids elicited in common only 10% of upregulated genes and 8% of downregulated genes (Figure 3). Although profiles elicited by a particular caterpillar species clustered together (Figure 1), responses of specific genes elicited were species-specific to a large extent (Figure 3) with only 21 and 12% of up- and down-regulated genes shared, respectively. Expression profiles elicited by the two aphids shared only 7 and 4% of all upregulated and downregulated genes, respectively. Caterpillars elicited roughly 3 times more upregulated genes and twice as many downregulated genes as the mechanical wounding. The amount of overlap between responses to caterpillars and mechanical wounding was similar to that observed between caterpillars and aphids (Figure 3).

The fewest transcriptional differences in response to caterpillars occurred between leaf types (attacked or “local” and unattacked or “systemic”) (Figure 2B). More genes were differentially expressed in leaves attacked by *S. exigua* compared with unattacked leaves on the same plant, whereas *P. rapae* differentially expressed roughly equal numbers of genes in both leaf types. Overlap in genes differentially expressed in the leaf type by the caterpillars also differed, with 19 and 9% overlap in local leaves vs. 16 and 13% overlap in systemic leaves for upregulated and downregulated genes, respectively. Data from both caterpillar species was combined by leaf type and compared to leaves wounded locally and systemic unwounded leaves. Few of the same genes elicited by caterpillars in the attached and unattacked leaves were elicited in both wounded and unwounded leaves at the same time points; 11–16% of upregulated and 2–15% of downregulated genes were common depending on caterpillar species leaf type and sampling time.

Transcriptional responses of *Arabidopsis* to herbivores were related to the insects' dietary breadth. The two dietary generalists upregulated more genes than did their respective dietary specialists (*p* < 0.0219, Supplemental Table 5). There was no difference (*p* > 0.2715) between them for downregulated genes.

### Transcriptional profiles were enriched in stress responses and shifts in primary metabolism

The DAVID Functional Clustering Tool was used to identify biological functions significantly enriched among differentially expressed genes. About two thirds of the genes differentially expressed in response to our treatments have some functional or structural information in TAIR. DAVID used TAIR and added information from other species for genes with little annotation in TAIR. Although most of these ontological data were collected in contexts other than responses to insects, we can use them to infer some functions of proteins encoded by genes influenced by our treatments.

Transcriptional responses to herbivory overlapped broadly with responses to 14 other biotic and abiotic stressors and stimuli, including responses to wounding, pathogens, cold, starvation, nutrient supply, metal ions, osmotic stress, reactive oxygen species, radiation, and light quantity and quality (Table 2). Caterpillars elicited the greatest number of different stress/stimulus responses, including many that were not elicited by wounding or aphids at either time point. *Pieris rapae* elicited transcriptional changes in 8 and *S. exigua* in 10 stimulus pathways linked to our treatments by DAVID at least once; each species elicited only two changes by the 6 h sampling. The two aphid species elicited fewer stress-related responses than did the caterpillars. *Brevicoryne brassicae* elicited changes in 4 stress-related pathways while *M. persicae* altered expression in 6 of the 14 relevant pathways. The transcriptional gene set most often altered by all the insects was response to hexose stimulus (6 of 16 possible interactions), but no single response gene set in this analysis characterized responses to insect elicitation as different from wounding or other stressors.

**Table 2:** Plant treatment-specific enrichment^a^ of differentially expressed genes within functional GO groups for biological processes associated with abiotic and biotic factors (^*^p ≤ 0.05 for modified Fisher's exact test, ^**^p ≤ 0.05 with Benjamini-Hochberg adjustment for multiple comparisons).

**Biological process GO annotation**	**Plant treatment**																
			**6 h local**			**6 h systemic**			**24 h Local**			**24 h systemic**			**6 h**		**24 h**	
**Identity**	**Type of process**	**Pr**	**Se**	**Wo**	**Pr**	**Se**	**Wo**	**Pr**	**Se**	**Wo**	**Pr**	**Se**	**Wo**	**Bb**	**Mp**	**Bb**	**Mp**
0006955	Immune response											^**^		^*^		^**^	^**^
0050832	Defense response to fungus			^*^		^*^										^**^	
0009617	Response to bacterium	^*^				^**^	^*^										
0009409	Response to cold								^**^			^**^				^**^	
0009267	Cellular response to starvation								^**^								
0031667	Response to nutrient levels							^*^	^*^								
0009746	Response to hexose stimulus	^*^		^*^					^*^		^*^	^*^			^*^		
0010038	Response to metal ion							^*^							^**^		^*^
0009651	Response to salt stress							^**^		^*^		^**^					
0006970	Response to osmotic stress							^*^	^*^			^**^					
0000302	Response to reactive oxygen						^*^		^*^						^**^		
0009314	Response to radiation							^*^					^*^		^**^		
0009642	Response to light intensity							^*^					^*^		^**^		
0010114	Response to red light													^*^			

*Abbreviations for plant treatment names are the same as those used in Table 1. See Supplemental Table 3 for annotation details*.

a *Functional enrichment was performed using the DAVID Functional Annotation Clustering and gene annotations of A. thaliana*.

Herbivory caused widespread changes in expression of genes involved in primary metabolism (Table 3). Both caterpillars and aphids elicited changes in expression of genes associated with metabolism of organic acids, fatty acids, lipids, and amino acids (Tables 3A–C). Expression of genes in metabolic pathways related to 12 amino acids was altered at least once by our treatments. *P. rapae* elicited changes in 11 of the 12; no other insect treatment elicited changes in more than five. Both caterpillars and aphids elicited changes in expression of genes associated with cell wall metabolism, although their impacts were different (Table 3D; Supplemental Figure 4). Aphids upregulated five times as many cell wall-related genes as did caterpillars, including a large number encoding extensins and extensin-like proteins, several peroxidases, and many cell wall-associated heat shock proteins and cognates. In contrast, caterpillars downregulated twice as many cell wall genes as did aphids, including those encoding galactosidases, xylosidases, and pectinases.

**Table 3:** Plant treatment-specific enrichment^a^ of differentially expressed genes within functional GO groups for biological process associated with metabolic processes (*p ≤ 0.05 for modified Fisher's exact test, **p ≤ 0.05 with Benjamini-Hochberg adjustment for multiple comparisons).

**Biological process GO annotation (identity~name)**	**6 h local**			**6 h systemic**			**24 h local**			**24 h systemic**			**6 h**		**24 h**	
	**Pr**	**Se**	**Wo**	**Pr**	**Se**	**Wo**	**Pr**	**Se**	**Wo**	**Pr**	**Se**	**Wo**	**Bb**	**Mp**	**Bb**	**Mp**
**A. ORGANIC ACID METABOLISM**																
0016053~organic acid biosynthesis	^**^	^*^			^**^		^**^	^**^		^**^				^**^	^*^	
0016054~organic acid catabolism							^*^	^*^		^*^				^**^		
**B. FATTY ACID AND LIPID METABOLISM**																
0006631~fatty acid metabolism	^**^		^*^		^**^					^**^				^**^	^*^	
0008610~lipid biosynthesis										^**^				^*^		
0016042~lipid catabolism														^*^		
**C. AMINO ACID METABOLISM**																
0008652~cellular amino acid biosynthesis	^*^	^*^						^*^	^*^	^*^				^*^		^*^
0009063~cellular amino acid catabolism	^*^							^*^		^*^				^**^		
0006525~arginine metabolism	^*^	^*^			^*^											
0006527~arginine catabolism	^*^															
0009068~aspartate amino acid catabolism														^*^		
0006534~cysteine metabolism								^*^		^**^						^*^
0019344~cysteine biosynthesis								^*^		^*^						^*^
0009065~glutamine amino acid catabolism	^*^															
0009069~serine amino acid metabolism										^*^						^*^
0009070~serine amino acid biosynthesis										^*^						^*^
0000162~tryptophan biosynthesis	^*^	^*^														
0009073~aromatic amino acid biosynthesis	^*^	^*^														
**D. CELL WALL METABOLISM**																
0042545~cell wall modification	^*^	^**^						^*^								
0052386~cell wall thickening		^*^			^*^					^*^				^*^		
0052543~callose deposition in cell wall		^*^						^*^		^*^				^*^		
0005199~structural constituent of cell wall													^**^	^**^		^**^
**E. CARBOHYDRATE METABOLISM**																
0016051~carbohydrate biosynthesis										^*^				^**^		^*^
0016052~carbohydrate catabolism														^*^		^*^
0005996~monosaccharide metabolism														^*^		
0046351~disaccharide biosynthesis														^**^		^*^
0009312~oligosaccharide biosynthesis														^**^		
0000271~polysaccharide biosynthesis											^*^			^*^		
0005992~trehalose biosynthesis														^*^		
**F. CHLOROPLAST**																
0009941~chloroplast envelope														^*^		
0009526~plastid envelope														^*^		
0009532~plastid stroma														^**^		
0009579~thylakoid														^*^		
**G. RESPONSE TO PLANT HORMONES**																
0009753~response to jasmonic acid stimulus	^**^	^**^	^**^	^**^	^**^	^**^	^**^		^*^	^**^		^*^				
0009751~response to salicylic acid stimulus									^*^					^*^		
0009723~response to ethylene stimulus								^*^			^*^					^*^
0009733~response to auxin stimulus	^*^			^*^	^*^											
0009735~response to cytokinin stimulus													^*^			
0009737~response to abscisic acid stimulus							^*^							^*^		
0009739~response to gibberellin stimulus	^*^	^*^														

*Abbreviations for plant treatment names are the same as those used in Table 1*.

a *Functional enrichment was performed using the DAVID Functional Annotation Clustering and gene annotations of A. thaliana*.

Aphids, but not caterpillars, caused changes in the expression of genes associated with carbohydrate metabolism and chloroplasts, and these changes were almost exclusively in response to *M. persicae* feeding at 6 h (Tables 3E,F, 4). *Myzus persicae* downregulated four genes for trehalose-6-phosphate synthases (TPS), two of which are known to influence levels of trehalose-6-phosphate *in vivo*; when reduced in *TPS* mutants, starch accumulation is inhibited and sugar phosphates accumulate (Table 4A; Baena-Gonzalez, 2010). This effect of TPS is thought to be mediated by downregulation of SnRK1, a protein kinase energy sensor that serves as a hub for generalized stress signaling. Although the SnRK1 gene itself was not differentially expressed in our experiments, *M. persicae* feeding at 6 h downregulated the expression of 9 of 24 known targets of SnRK1 (Supplemental Figure 5; Baena-Gonzalez and Sheen, 2008).

**Table 4:** **Plant treatment-specific enrichment^a^ of differentially expressed genes associated with the acquisition and turnover of carbon and nitrogen**.

***A. thaliana* gene locus**	**6 h local**			**6 h systemic**			**24 h local**			**24 h systemic**			**6 h**		**24 h**		**Gene symbol**
	**Pr**	**Se**	**Wo**	**Pr**	**Se**	**Wo**	**Pr**	**Se**	**Wo**	**Pr**	**Se**	**Wo**	**Bb**	**Mp**	**Bb**	**Mp**	
**A. TREHALOSE SYNTHASES**																	
AT1G70290	−0.4	−0.4	0.2	−0.4	−0.3	−0.2	−1.2	−0.6	0.4	−0.4	−0.8	0.6	0.0	**−2.1**	**1.7**	0.2	TPS8
AT1G23870	0.0	−0.1	−0.1	−0.1	−0.8	0.0	−0.6	−0.1	−0.1	0.5	−0.4	−0.2	0.2	**−2.0**	0.3	−0.4	TPS9
AT1G60140	−0.1	0.3	−0.2	−0.7	−0.3	0.8	−1.3	−0.5	−0.8	−0.8	−0.8	0.4	−0.3	**−1.8**	0.5	−0.4	TPS10
AT2G18700	0.8	−0.2	−0.6	0.7	−0.5	−0.2	−0.5	−0.5	0.6	−0.8	−0.8	0.3	−0.4	**−1.8**	0.7	−0.5	TPS11
**B. CALVIN CYCLE**																	
AT1G42970	0.9	−0.3	0.2	0.3	−0.6	−0.4	0.4	0.6	0.4	−0.9	−0.7	0.3	0.2	**1.1**	−1.0	−0.2	GAPB
AT3G55800	0.1	−0.7	−0.7	−1.0	−0.1	−0.5	0.4	0.3	0.3	0.2	0.2	0.5	0.1	**1.1**	−0.4	0.7	SBPase
AT1G32060	0.0	−0.3	−0.6	−0.1	−0.3	−0.3	0.7	0.1	−0.3	0.4	0.3	0.4	−0.1	**1.2**	0.1	0.4	PRK
AT2G21330	−0.4	−1.1	0.6	0.4	0.1	−0.3	−0.1	0.7	−0.1	−1.0	−0.4	−0.6	0.1	**1.1**	−0.3	−0.4	FBA1
AT3G60750	0.0	0.2	0.0	−0.4	0.5	0.0	−0.8	0.3	0.3	0.2	−0.1	−0.1	0.3	**1.6**	0.3	0.5	TK
AT1G71100	0.6	0.6	0.2	0.0	0.2	0.7	0.1	0.6	0.3	0.5	0.2	−0.1	0.3	**1.8**	0.2	0.5	RSW10
**C. AMINO ACID DEGRADATION**																	
AT1G08630	−0.4	−1.0	−0.3	−0.6	−0.1	0.3	0.4	0.6	0.2	1.1	−0.5	0.2	1.2	**−1.1**	−0.2	−0.7	THA1
AT1G64660	0.5	0.6	−0.2	−0.3	0.3	1.6	0.1	0.7	0.8	0.5	0.4	−0.2	0.0	**−2.4**	−0.3	−0.9	MGL
AT4G33150	0.7	−0.2	0.4	0.4	0.4	1.9	−1.2	0.9	−1.3	−0.5	0.3	0.0	0.5	**−1.3**	0.1	−1.8	LKR=SDH
AT1G03090	0.9	−1.0	0.0	−0.1	−0.1	0.4	−0.9	−0.4	−0.3	−1.4	−0.3	−1.0	0.0	**−1.4**	−0.3	−1.4	MCCA
AT5G54080	0.3	0.5	−0.2	0.3	−0.3	0.1	−0.8	−0.1	0.4	−0.2	−0.1	−0.1	−0.3	**−1.1**	0.0	−0.5	HGO
**D. NITROGEN ASSIMILATION, ASPARAGINE SYNTHESIS, REGULATION OF PROTEIN SYNTHESIS**																	
At5g04140	0.2	−0.2	−0.4	0.4	0.0	−0.9	0.5	0.0	−0.6	0.5	0.3	−0.2	0.0	**1.3**	−0.6	0.5	GLU1
At3g47340	−0.1	0.8	**−1.3**	−0.8	0.8	0.1	**−2.4**	**−1.9**	**−1.4**	**−2.1**	−1.5	−0.7	−0.5	**−4.4**	0.0	−1.5	ASN1/DIN6
At5g09930	−0.4	0.1	−0.1	−0.1	−0.2	−0.4	0.3	0.1	−0.1	0.0	0.2	0.0	0.6	**1.2**	0.4	0.0	GCN2

*Abbreviations for plant treatment names are the same as those used in Table 1. Values are fold change and those in boldface are significant*.

a *Functional enrichment was performed using the DAVID Functional Annotation Clustering and gene annotations of A. thaliana*.

*Myzus persicae* uniquely upregulated a suite of Calvin Cycle genes at 6 h (Table 4B). These included glyceraldehyde-3-phosphate dehydrogenase (GAPB), sedoheptulose-1,7-bisphosphatase (SBPase), fructose-bisphosphate aldolase (FBA1), phosphoribulokinase (PRK), phosphoglycerate kinase (PGK), ribose 5-phosphate isomerase (RBI), and transketolase (TK). Another suite of genes uniquely downregulated by *M. persicae* at 6 h are involved in amino acid catabolism (Tables 3C, 4C). These included threonine aldolase (THA1), methionine gamma-lyase (MGL), lysine ketoglutarate reductase (LKR), methylcrotonoyl-CoA carboxylase activity (MCCA), which degrades leucine, and homogentisate 1,2-dioxygenase (Abbot and Withgott, 2004) which degrades l-phenylalanine and tyrosine (Table 4C). *Myzus persicae* strongly downregulated asparagine synthase 1 (ASN1) which makes asparagine by amidation of aspartate using glutamate or ammonium as the amide donor (Gaufichon et al., 2010). This gene was also downregulated by caterpillars and wounding but not to the extent that it was downregulated by aphids (Table 4D). *Myzus persicae* upregulated the expression of ferridoxin-dependent glutamate synthetase 1 (Fd-GOGAT1 or GLU1), a key enzyme in the assimilation of inorganic nitrogen and reassimilation of ammonia released by photorespiration, and the translational inhibitor GCN2, whose phosphorylation of eIF2-alpha reduces overall protein synthesis (Table 4D; Baena-Gonzalez, 2010; Hey et al., 2010; Kissen et al., 2010).

Caterpillars and aphids caused very different changes in the expression of genes associated with amino acid metabolism. Caterpillars, but not aphids, altered the expression of genes involved in biosynthesis of indole and aromatic amino acids (Table 3C). Caterpillars upregulated three genes directly involved in TRP biosynthesis, three involved in indole glucosinolate (GS) synthesis, and one in auxin biosynthesis (Tables 5A,B). One gene involved in indole GS synthesis was downregulated by both aphids at 24 h (Table 5B).

**Table 5:** **Plant treatment-specific enrichment^a^ of differentially expressed genes associated with tryptophan and glucosinolate biosynthesis**.

***A. thaliana* gene locus**	**6 h Local**			**6 h systemic**			**24 h local**			**24 h systemic**			**6 h**		**24 h**		**Gene symbol**
	**Pr**	**Se**	**Wo**	**Pr**	**Se**	**Wo**	**Pr**	**Se**	**Wo**	**Pr**	**Se**	**Wo**	**Bb**	**Mp**	**Bb**	**Mp**	
**A. TRYPTOPHAN BIOSYNTHESIS**																	
AT3G54640	**1.4**	**1.5**	0.1	−0.2	0.4	0.5	0.6	0.0	−0.3	**1.3**	0.2	−0.3	0.5	0.8	0.6	1.3	TRP3/TPA1
AT1G52410	**2.3**	**3.3**	**1.6**	**1.9**	**2.1**	1.5	1.1	1.3	0.4	**2.2**	0.9	0.3	0.2	−0.2	−0.7	0.8	TSA1
AT1G69370	0.1	1.2	−0.2	0.6	−0.4	−0.2	−0.1	−0.1	−0.3	0.9	0.4	0.2	−0.2	−0.4	−0.2	−0.5	CM3
**B. INDOLE GLUCOSINOLATE BIOSYNTHESIS**																	
AT4G31500	1.0	**1.4**	0.1	−0.4	0.6	0.5	0.8	**1.2**	0.3	0.8	0.7	−0.4	−0.7	0.7	0.6	1.0	cyp83B1/ATR4
AT5G60890	**1.9**	**1.2**	1.2	0.8	1.1	0.2	0.7	0.2	−0.9	0.3	0.4	−0.5	−0.7	0.1	**−1.5**	**−1.6**	MYB34/ATR1
AT4G39950	**1.7**	**1.6**	0.8	1.0	**1.5**	**1.0**	**1.7**	**1.4**	0.5	**1.2**	1.0	0.1	−0.4	**1.1**	1.0	1.1	cyp79B2
AT3G44300	−0.3	**1.2**	0.7	−0.4	0.1	0.0	−0.5	0.0	1.4	0.6	0.1	−0.5	0.5	**1.3**	−0.1	0.8	NIT2
**C. ALIPHATIC GLUCOSINOLATE BIOSYNTHESIS**																	
AT4G03050	**1.5**	1.1	0.1	0.4	0.6	0.7	**1.1**	0.8	0.0	**1.2**	0.8	0.0	0.2	0.9	−0.5	0.0	AOP3
AT2G25450	0.9	1.1	0.0	0.3	0.5	0.5	**1.1**	0.9	0.7	0.9	0.3	0.5	0.3	−0.4	0.6	0.9	
AT1G62560	0.5	0.5	0.5	0.8	0.8	0.7	**1.7**	1.0	0.5	1.0	0.0	0.7	0.1	**1.3**	0.3	1.4	FMO GS-OX3
AT3G19710	0.9	0.4	0.6	−0.4	0.8	0.5	0.1	**1.1**	0.3	**1.5**	0.6	0.3	0.2	0.1	0.7	0.6	BCAT4
AT1G65860	0.4	0.5	0.0	0.2	0.5	0.1	1.0	**1.1**	0.1	0.6	0.1	0.4	−0.1	0.5	0.1	0.6	FMO GS-OX1
AT5G23010	1.3	**1.5**	0.9	0.2	0.7	0.7	0.3	0.6	−0.2	0.6	0.6	0.1	0.0	0.0	0.3	−0.2	MAM1
**D. BOTH INDOLYL AND ALIPHATIC GLUCOSINOLATE BIOSYNTHESIS**																	
AT2G20610	0.8	0.7	−0.2	0.5	0.5	0.4	**1.3**	0.6	0.3	1.7	0.9	0.3	0.6	**1.2**	0.4	0.8	C-S lyase
AT1G74090	0.6	**1.0**	0.4	0.7	0.9	0.4	1.0	0.8	0.0	**1.0**	0.9	0.1	0.2	1.8	0.3	0.9	SOT18
**E. NITRILE FORMATION (GLUCOSINOLATE TO NITRILE INSTEAD OF CYANATE)**																	
AT3G16410	**1.7**	**1.6**	0.7	0.8	0.4	1.3	0.1	0.8	0.3	1.1	0.4	−0.2	0.0	0.1	−0.5	−0.6	NSP4
AT3G16400	**1.6**	**2.0**	1.2	**1.4**	**1.2**	1.1	0.4	0.7	−0.6	1.0	−0.7	−0.2	−0.5	0.2	−0.3	1.0	NSP1
AT3G16390	**1.7**	**2.7**	**1.0**	0.6	0.6	1.0	0.4	0.2	0.3	0.3	−0.1	0.1	−0.3	0.4	−0.8	0.1	NSP3

*Abbreviations for plant treatment names are the same as those used in Table 1. Values are fold change and those in boldface are statistically significant*.

a *Functional enrichment was performed using the DAVID Functional Annotation Clustering and gene annotations of A. thaliana*.

Consistent with upregulation of genes involved in biosynthesis of amino acids from which GS are derived, caterpillars also uniquely influenced expression of genes specifically associated with GS biosynthesis and activity (Table 3D). These included a wide variety of genes in nitrogen and sulfur assimilation and modulation of GS activity (Supplemental Table 6). Responses to caterpillars were functionally enriched in genes involved in glucosinolate metabolism; responses to aphids were not (Tables 3G, 5). Genes involved in glucosinolate biosynthesis were upregulated by *S. exigua* in damaged leaves at both time points and by *P. rapae* at 24 h in both leaf types (Tables 5B–D). Genes involved in glucosinolate catabolism, e.g., nitrile biosynthesis genes that promote simple nitrile, but not epithionitrile or thiocyanate formation, were upregulated by both caterpillar species in damaged tissue at 6 h (Table 5E). Aphids failed to alter the expression of enough glucosinolate-related genes to generate a statistically significant enrichment pattern.

### Transcriptional profiles were enriched in genes associated with hormone signaling

**Figure 4:**
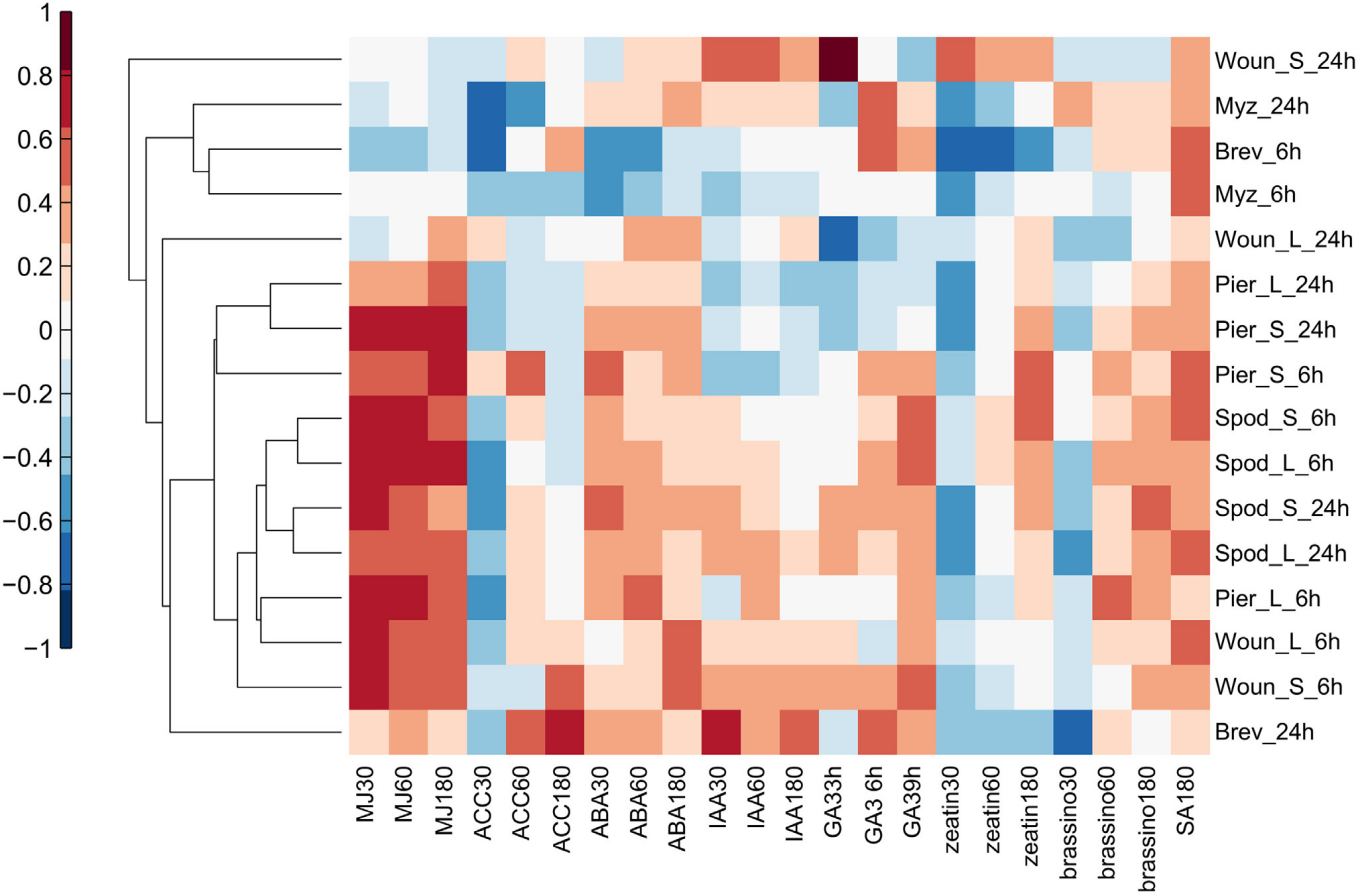
**Hormonometer analysis of differential gene expression by *A. thaliana* in response to insect feeding and mechanical wounding**. The response in gene expression of *Arabidopsis* in our treatments is compared with that of *Arabidopsis* at 30, 60, and 180 min, or 3, 6, and 9 h, after hormone application. The magnitude of correlation in gene expression is indicated by the color scale at bottom and correlation values of 0.4 and above are considered statistically significant. Treatment abbreviations are the same as in Table 1. MJ, methyl jasmonate; ACC, 1-aminocyclopropane-1-caroxylic acid; ABA, abscisic acid; IAA, indole-3-acetic acid; GA3, gibberellic acid 3; zeatin, cytokinin; brassino, brassinosteroid.

To determine whether particular hormone signaling pathways were involved in responses to insect attack, the Hormonometer Tool was used to evaluate the similarity in expression profiles elicited by insects to those elicited by exogenous application of plant hormones. The early, 6 h wound treatments and all of the caterpillar treatments and times strongly upregulated genes associated with responses to methyl jasmonate (MeJA) in all leaf types (Figure 4). There was some evidence that the aphid *B. brassicae* downregulated MeJA-related responses.

Caterpillar treatments (but not wounding or aphids) elicited expression of genes associated with responses to abscisic acid (ABA) in all leaf types at both times (Figure 4). All of the insect treatments tended to downregulate genes associated with early responses to ethylene and cytokinins and upregulated genes associated with salicylic acid (SA; Figure 4). These patterns were generally supported by the DAVID analysis (Table 3G) although that analysis found fewer significant associations.

### Transcriptional profiles induced by caterpillars differed in local and systemic leaves

We used the DAVID tool to examine functional differences between transcriptional responses of two leaf types; leaves damaged directly (“local”) by caterpillars and age-matched undamaged (“systemic”) leaves on the same plants. DAVID identified 8 functional categories that were significantly enriched in one or more combinations of leaf type, caterpillar and time (Table 6, Supplemental Table 6). Two of these were found in a single combination; response of genes involved in water deprivation were upregulated only at 6 h in leaves damaged by *S. exigua* and responses of genes associated with lectins were upregulated only at 24 h in leaves damaged by *P. rapae*. Ethylene signaling pathway genes were upregulated in both local and systemic leaves only at 24 h by *S. exigua*. Responses of genes involved in sulfur metabolism were upregulated at both time points in leaves damaged by *S. exigua*. Both species elicited responses in genes involved in auxin signaling at 6 h, but in different leaf types, systemic for *P. rapae* and local for *S. exigua*. Most of the auxin signaling-related genes in the enriched functional category were downregulated by insects (Supplemental Table 6).

**Table 6:** **Functional enrichment^a^ of genes differentially expressed in only local leaves, only systemic leaves, or both local and systemic leaves when asterisks span these treatment columns within an insect and time**.

**Biological process GO annotation (identity~name)**	**Pr-L 6 h**	**Pr-S 6 h**	**Se-L 6 h**	**Se-S 6 h**	**Pr-L 24 h**	**Pr-S 24 h**	**Se-L 24 h**	**Se-S 24 h**
0009611 ~ response to wounding						^***^		
	^****^		^****^				^****^	
0032260 ~ response to JA stimulus	^****^		^****^				^***^	
0031408 ~ oxylipin metabolism	−/^*^			−/^*^		^*^/−		
0009733 ~ response to auxin		^****^/^**^	^*^/−					
0009873 ~ ethylene-activated signaling pathway							^*^	
0006790 ~ sulfur compound metabolism			^**^				^***^	
0009414 ~ response to water deprivation							^**^	

Asterisks in clear/white spaces denote the enrichment Benjamini-Hochberg p-values: ^*^p < 0.05, ^**^ p < 0.01, ^***^p < 0.005, ^****^p < 0.001, ^*****^p < 0.0005, ^******^p < 0.0001. Dashes indicate no functional enrichment for one partner in the pair of terms. Abbreviations for plant treatment names are the same as those used in Table 1.

a *Functional enrichment was performed using the DAVID Functional Annotation Clustering and gene annotations of A. thaliana*.

**Figure 5:**
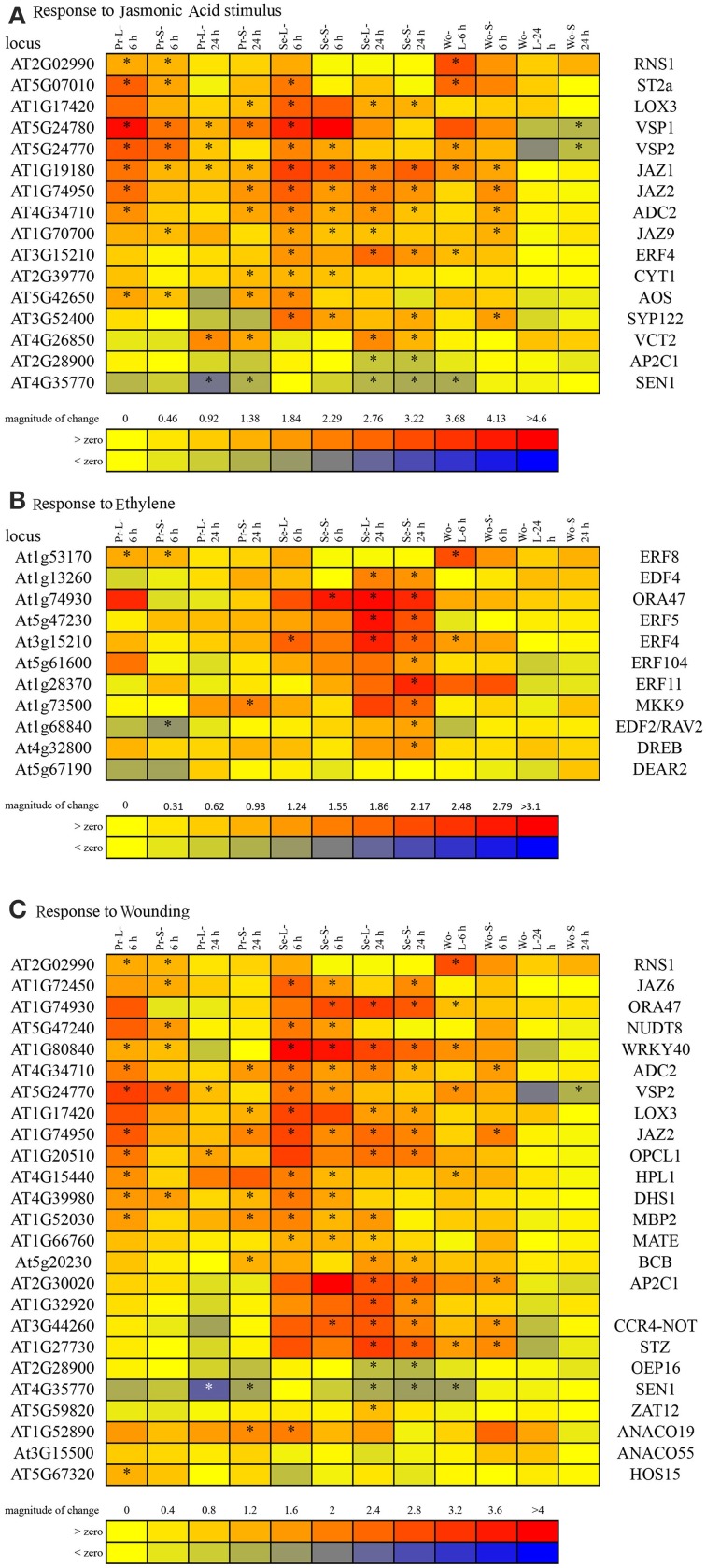
**Differential expression of genes enriched in both leaf types (local and systemic) of the caterpillar treatments and associated with responses to: (A) Jasmonic acid stimulus, (B) Wounding, and (C) ethylene**. Asterisks indicate treatments for which gene expression was significantly different from controls. Abbreviations for plant treatments as in Table 1.

Both caterpillars elicited expression of genes associated with responses to wounding and jasmonates in all leaf types at 6 h (Table 6, Supplemental Table 6). Oxylipin biosynthesis/metabolism-related responses occurred only in local leaves at 6 h for *P. rapae* compared to only in systemic leaves at both times for *S. exigua*. Wound-typical, other than JA responses, continued into the 24 h sampling of systemic leaves in *P. rapae* treatments. JA- and wound-related responses comprised upregulation except for two genes, which were downregulated by both caterpillar species in all leaves at 24 h (Supplemental Table 6). Genes associated with responses to both wound and JA continued to be elicited in all leaves at 24 h in *S. exigua* treatments. Expression of a subset of jasmonate- and wound-responsive genes was consistently upregulated by *S. exigua*, but not *P. rapae*, at both time points, including *ERF4 (AT3G15210), ORA47 (AT1G74930), WRKY40 (AT1G80840), AP2C1 (AT2G28900), CCR-4-NOT (AT3G44260), SYP122 (AT3G52400), and STZ (AT1G27730)* (Figures 5A,B). This duration of ethylene-responsive *ERF4* and *ORA47* expression implicates ethylene signaling. Indeed, both leaf types at 24 h for *S. exigua* elicited expression of genes associated with the ethylene signaling pathway at higher levels than did *P. rapae* (Figure 5C).

### Coexpression network analysis identified modules of coexpression and putative hub genes

The weighted gene coexpression analysis identified a non-random network architecture of 18 modules of coexpressed genes (Supplemental Figure 4a). All but one of the modules was statistically associated with treatments, and there was no overlap in modules responding to caterpillars, mechanical wounding, and aphids (Table 7). For caterpillars, the “cyan,” “blue,” “light yellow” and “tan” modules were associated with responses to *P. rapae*, whereas the “light cyan,” “green yellow,” “red,” “gray60” and “dark red” modules were associated with responses to *S. exigua*. Only one of the modules (turquoise) was associated with mechanical wounding (local and systemic responses at 6 h). For aphids, the “yellow,” “salmon” and “brown” modules were associated with responses to *B. brassicae*, whereas the “black,” “dark turquoise,” “midnight blue,” “brown” and “dark green” modules were associated with responses to *M. persicae*.

**Table 7:**
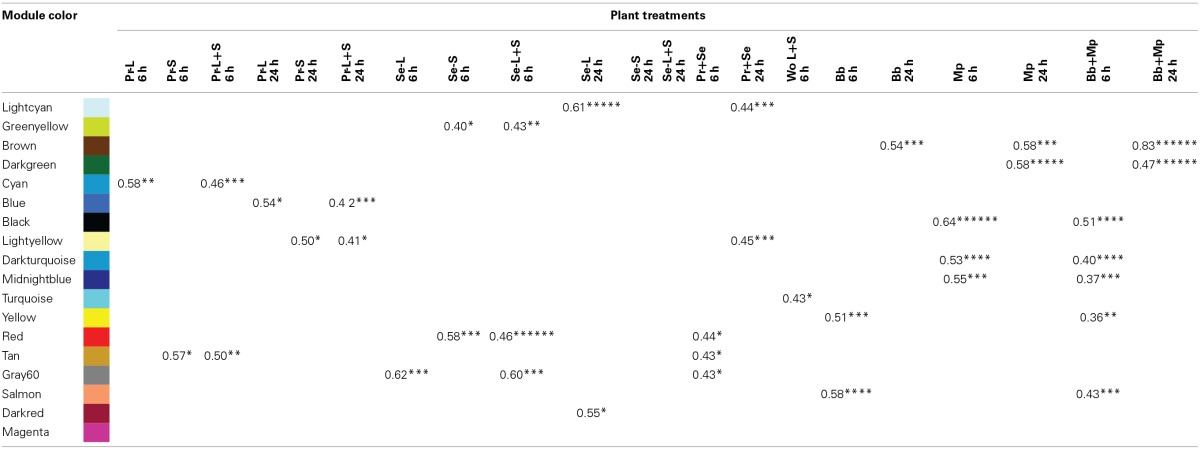
**Network correlation modules of differentially expressed genes coexpressed according to plant treatment**.

Only modules with eigenvalues > 0.35 were considered significantly associated with a treatment. Asterisks denote the p-value for the given eigenvalue: ^*^p < 0.05, ^**^p < 0.01, ^***^p < 0.005, ^****^p < 0.001, ^*****^p < 0.0005, ^******^p < 0.0001. Abbreviations for plant treatment names are the same as those used in Table 1.

We then looked to see if any species-specific modules were also characteristic of caterpillars or aphids in general. The “red,” “tan” and “gray60” modules were associated with 6 h responses to caterpillars, and the “light cyan” and “light yellow” modules were associated with 24 h responses to caterpillars. The “black,” “dark turquoise,” “midnight blue,” “yellow” and “salmon modules were associated with 6 h responses to aphids,” and the “brown” and “dark green” modules were associated with the 24 h responses to aphids. These patterns are reflected in the three main arms of the network architecture, comprising the 6 h responses to wounding (“turquoise”), caterpillars (“red”), and aphids (“midnightblue”) (Supplemental Figure 4b).

Patterns of gene function within modules were determined by examining the ontological enrichment of their member genes. Only a few of the 17 modules associated with treatments were enriched in specific gene functions, which may reflect the absence of functional annotations. To identify regulatory hubs that may direct treatment-specific responses, only module membership values ≥0.8 were used to recognize highly connected module members. These results are summarized for all modules in Supplemental Table 7. Although all modules contained one or more highly connected genes, only a few of those genes are characterized, and are discussed below. A majority of the potential hub genes are either of unknown function or have only an hypothesized function based on sequence similarity.

The “green yellow” module was statistically associated with responses to *S. exigua* at 6 h in unattacked, systemic leaves (Table 7). Ontological analysis indicated that this module was enriched in genes associated with responses to JA, bacteria, and chitin (Supplemental Table 8). Highly connected genes in this module were examined for their potential to serve as hub genes directing treatment-specific plant responses, and four (At1g80840, At5g54145, At5g35475, At1g57990) had module memberships ≥ 0.8 suggesting that they have potential regulatory importance. WRKY40 (At1g80840) is a transcription factor that binds W-box sequences and forms protein complexes with itself and with WRKY18 and WRKY60 to influence *Arabidopsis* susceptibility to biotic and abiotic stresses (Xu et al., 2006; Chen et al., 2012). The gene At5g54145 is an expressed protein of unknown function, while At5g35475 is similar to a hAT dimerization domain-containing protein/transposase-related. The At1g57990 gene is related to the purine transporter PUP1, which may be involved in the transport of purines and purine derivatives like cytokinins across the plasma membrane.

To infer the functions of these potential hub genes, we used ATTED to look for gene subnetworks they anchor. However, only WRKY40 was present on the Affymmetrix arrays used to construct the ATTED network (Obayashi and Kinoshita, 2010). In this network, WRKY40 interacts with three genes identified with plant responses to wounding (CCR4-NOT, AT3G44260; BCB, At5g20230; RHL41/ZAT1, AT5G59820) and two genes identified with plant responses to ethylene (ERF11, At1g28370; ACS6, At4g11280) (Supplemental Figure 5). WRKY40 links many such genes elicited by *S. exigua* but not the other insects in our study, and is thus a candidate hub gene.

The network coexpression analysis also identified several other potential hub genes for early *Arabidopsis* responses to *M. persicae*. Two modules (“dark turquoise” and “black”) were statistically associated with responses to *M. persicae* at 6 h (Table 7). The dark turquoise module was enriched in genes associated with extensin-like activity, including many of the cell wall associated genes upregulated by *M. persicae* at 6 h (Supplemental Figure 4, Supplemental Table 8). There were two highly connected, potential hub genes in the dark turquoise module, a calcium-binding EF-hand family protein (At3g47480) about which little is known, and VBF1 (At1g47056), a VIER F-box protein known to be a positive regulator of auxin response and cell wall metabolism (Supplemental Table 8; Schwager et al., 2007). Although there was no functional enrichment of all of the genes in the black module, the potential hub genes were enriched in an annotation of “extracellular region.” These genes included a putative peroxidase (At4g37520), two disease resistance proteins (SOBIR1, At2g31880; CC-NBS-LRR, At4g33300) and three genes upregulated by *M. persicae* at 6 h and associated with cell wall metabolism (Supplemental Figure 4; EXP4, At2g39700; HSP81.4, At5g56000; GUS2, At5g07830).

### *Cis*-elements of insect regulated genes explain only some of the transcriptional differences

**Figure 6:**
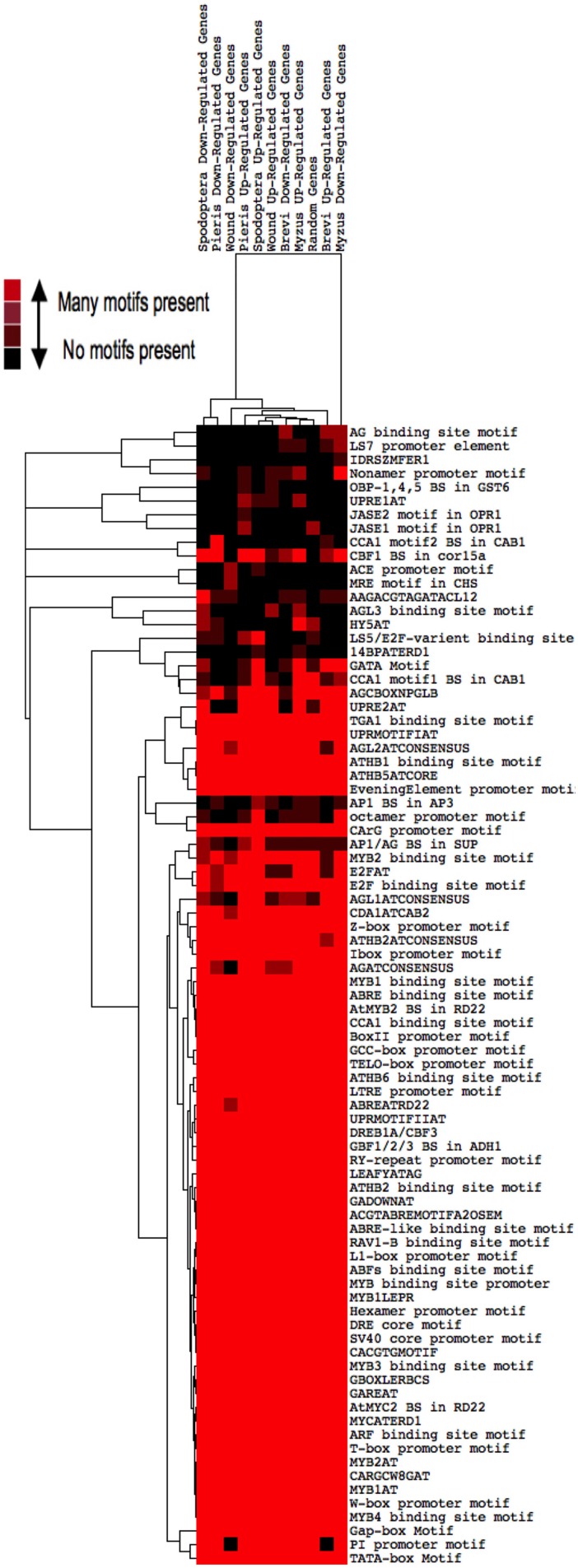
**Heat map of *Cis*-element distributions in genes differentially expressed by insect herbivory and mechanical wounding**. Transcription factor binding sites up to 1000 bp upstream of all differentially expressed genes were located using the PLACE database and clustered by treatment. Copies of a motif found is indicated by the color scale; e.g., red, many; black, none.

We conducted three separate analyses to identify potential *cis*-elements that are involved in TF signaling pathways responding to our treatments. First, we found that most genes whose expression was altered by insect feeding shared the great majority of known or postulated TF binding sites (Figure 6). A cluster analysis found that the great majority of these genes contained all of the known *cis*-elements. However, 2 clades appeared distinct. The *cis*-element composition of genes down-regulated by *M. persicae* formed a single clade, and c*is*-regulatory sequences of genes down-regulated by the two caterpillars, *S. exigua* and *P. rapae*, comprised another. *Cis*-elements of genes involved in responses to wounding did not form a distinct clade.

ATHENA enabled identification of eight *cis*-elements that were over-represented (*p* < 0.00001) in genes co-expressed in response to individual treatments (Table 8). These elements may serve as important regulatory components of signaling pathways responding to insect attack. Several of the enriched *cis*-elements belong to well characterized classes, including ABREs, G-Boxes, TATA boxes, WRKY boxes, and I-boxes. TATA boxes were over-represented in many of the genes upregulated by caterpillars, while I-boxes, found in many light-regulated genes (Giuliano et al., 1988), were enriched in genes downregulated by caterpillars. Interestingly, most treatments repressed genes that were enriched in “*Evening Element*” motifs. This *cis*-element is characteristic of many genes whose expression peaks at dusk (Harmer et al., 2000).

**Table 8:** ***Cis*-elements enriched^a^ in genes differentially expressed by insect herbivory and mechanical wounding**.

**Motif name**	**Motif function**	**Motif sequence**	**Insect**	**Direction of change**	**Hours after feeding**	**Leaf type**	***p*-Value**	**Y/N^b^**	**References**
ABRE-like binding site	ABA-responsiveness	BACGTGKM	*B. brassicae*	Up	24	All	1.00E-06	Y	Shinozaki and Yamaguchi-Shinozaki, 2000
			*M. persicae*	Down	6	Whole Plant	1.00E-05	Y	
ACGT/ ABRE MOTIF	ABA-responsiveness	ACGTGKC	*P. rapae*	Up	24	All	1.00E-05	Y	Hattori et al., 2002
AtMYC2 MOTIF	AtMYC2 Binding/dehydration response	CACATG	*S. exigua*	Up	24	Systemic	1.00E-05	N	Abe et al., 1997
CACGTGMOTIF	AtGBF4 Binding	CACGTG	*B. brassicae*	Up	24	All	1.00E-07	Y	Chakravarthy et al., 2003
			*P. rapae*	Up	24	Systemic	1.00E-05	Y	
Evening Element	Circadian rhythms	AAAATATCT	*B. brassicae*	Up	24	All	1.00E-05	N	Harmer et al., 2000
			*M. persicae*	Down	6	All	1.00E-10	N	
			*P. rapae*	Down	24	Systemic	1.00E-06	N	
			*S. exigua*	Down	24	Local	1.00E-05	N	
			*S. exigua*	Down	24	Systemic	1.00E-05	N	
Ibox	Light -regulated gene expression	GATAAG	*M. persicae*	Down	6	All	1.00E-05	N	Giuliano et al., 1988
			*P. rapae*	Down	24	Systemic	1.00E-05	N	
TATA-box			*S. exigua*	Up	24	Local	1.00E-10	N	Basehoar et al., 2004
			*S. exigua*	Up	6	Local	1.00E-07	N	
			*P. rapae*	Up	24	Systemic	1.00E-06	N	
			*P. rapae*	Up	6	Local	1.00E-05	N	
			*S. exigua*	Up	6	Systemic	1.00E-05	N	
			*S. exigua*	Up	24	Systemic	1.00E-05	N	
WBOX MOTIF	Binding site for WRKY proteins and SA-responsiveness	TTTGACT	*S. exigua*	Up	24	Local	1.00E-05	N	Yu et al., 2001
			*S. exigua*	Up	24	Systemic	1.00E-05	Y	
									

a *Enriched signifies that numbers of a motif found in each set of treatments are higher than would randomly be found in the genome (p < 1.0^e − 5^; ATHENA)*.

b *Motifs confirmed using the Motif Sampler exhaustive search tool are noted with a Y = confirmed and N = not confirmed*.

We also conducted a *cis*-element analysis using the MotifSampler tool, which unlike ATHENA, can detect enrichment of both unknown and known motifs (Supplemental Table 9) (Thijs et al., 2001, 2002). However, there was a high nucleotide substitution rate due to sequence consensuses, so we wrote a script that resolved all degenerate nucleotide sequences, predicted all possible 6-, 7-, and 8-mers identified by MotifSampler, and then matched them against the Athena *cis*-element database. This process enabled us to confirm 6 of the 20 putative elements identified by MotifSampler and relate them to our ATHENA results (Table 8). The reason why there is not 100% confirmation is due to differences in methodology between MotifSampler and ATHENA. Motif Sampler tries to find over-represented motifs in the upstream region of a set of co-regulated genes. This type of motif finding-algorithm uses Gibbs sampling to find the position probability matrix that represents the motif and uses background models to improve the robustness of the motif finding. Therefore, if no significant difference exists between background and entered list, the motifs will not be predicted due to lack of higher scores. Nonetheless, these motifs would have to be experimentally verified to confirm their role in insect responses.

## Discussion

### Sources of specificity in plant responses to herbivores and wounding

Transcriptional responses to insects are distinct from responses to wounding in both number and identities of responsive genes. Here we focused our analysis on gene enrichment to identify changes in gene expression of groups of functionally related genes, to address the limitations of microarray technology (false positives and false negatives) which require that expression changes of individual genes be confirmed. The larger number of genes upregulated by the two dietary generalists is consistent with the commonly held hypothesis that dietary specialists have evolved a stealthier way to exploit their host plants than dietary generalists (Ali and Agrawal, 2012). But, as others have noted, it is unwise to draw conclusions from studies such as ours that lack many examples of insects of each dietary type (Bidart-Bouzat and Kliebenstein, 2011).

Insect herbivores provide potential signals to plants that reveal their identity by feeding at different times of the day, on different kinds of plant tissue, on tissue of different ages, causing different damage patterns and amounts, and/or any chemistry they introduce with oral secretions (Bonaventure et al., 2011; Maffei et al., 2012). These signals generate HAMPs that plants can use to identify their attackers and respond appropriately. Evidently HAMPs set insects apart even though insect feeding may involve extensive wounding. Therefore, given their very different feeding styles, it is not surprising that aphids and caterpillars used in our study elicited changes in gene expression with only about 10% of genes in common in this study.

Aphids were given free range of the plant in our study and usually chose to feed on phloem of younger leaves. Although there was little difference between the two aphid species in feeding sites, about 90% of the genes whose expression they elicited were different from each other. Thus, the specific searching behavior of their stylets within the plants and the oral secretions they release are likely sources of differentiating signals (Will et al., 2012). Aphid stylets secrete a variety of proteins as they make their way through and around other cells in the leaf to reach the phloem. The list of proteins is extensive and includes lipases, peroxidases, pectinases, and glucosidases as well as many proteins of unidentified function (Miles, 1999; Elzinga and Jander, 2013). Several have been identified from *M. persicae*, but the paucity of functional studies makes it premature to suggest which may be involved in the different responses we observed.

In the caterpillar experiments, we kept tissue and age constant by restricting feeding to specific, similarly aged leaves and had caterpillars feed until they removed similar amounts of leaf area. Despite this, only a little more than 20 and 10% of upregulated and downregulated gene expression, respectively, were elicited in common by both species. The caterpillars exhibited some differences in feeding pattern, with *P. rapae* eating continuously in a single location, usually on a leaf margin, whereas *S. exigua* usually fed at several locations on the leaf and rarely from the margin. As a result, the observed differences in the effect of these two species on gene expression could have been caused by differences in their patterns of damage, their oral secretions deposited at the feeding site or both. Oral secretions of caterpillars are known to modulate plant responses to mechanical wounding. Fatty acid—amino acid conjugates (FACs) and glucose oxidase (GOX) are in the regurgitant of *S. exigua* (Alborn et al., 1997; Pohnert et al., 1999; Diezel et al., 2009). Although *P. rapae* is unstudied, its congener *P. brassicae* produces beta-glucosidases in regurgitant (Mattiacci et al., 1995). Expression of an ERF/AP2 transcription factor in *Arabidopsis* was suppressed by saliva from *S. littoralis* and *P. brassicae* caterpillars, which are congeneric with ours, but FACs were not the source of suppression (Consales et al., 2011). Considerably more work will be required to identify the many HAMPs likely present in diverse insect species.

### Stress responses and reconfiguration of metabolism

Expression of genes typical of responses to other stressors is a common feature of plant responses to herbivory. Most of these responses involve a reconfiguration of primary metabolism (Schwachtje and Baldwin, 2008). The changes we observed in the *Arabidopsis* transcriptome generally reflect downregulation of key metabolic components following insect attack. The detection of stress caused by insect feeding is thought to be mediated by SNF1/AMPK/SnRK1 protein kinases, which as energy sensors serve as hubs for generalized stress signaling (Polge et al., 2008; Crozet et al., 2014). In *Nicotiana attenuata*, a SnRKkinase regulates reallocation of photoassimilates in response to chewing herbivores (Schwachtje et al., 2006). SnRK1 also likely mediated downregulation of four TPS genes by *M. persicae* in our study. Although the SnRK1 gene itself was not differentially expressed in our experiments, *M. persicae* feeding at 6 h downregulated the expression of 9 of 24 known targets of SnRK1 (Supplemental Figure 3; Baena-Gonzalez and Sheen, 2008).

Downregulation of photosynthesis is a common response to biotic stress and has been previously reported to also occur in response to herbivory (Zangerl et al., 2002; Giri et al., 2006; Tang et al., 2006; Bilgin et al., 2010). Although we did not measure photosynthesis in our study, we found caterpillars and aphids to have different impacts on gene expression associated with photosynthesis, with *M. persicae* upregulating Calvin Cycle genes and the caterpillars having little effect.

Some of the plant transcriptional responses to insects involved hexoses. Previous work also has shown that chewing herbivores can stimulate localized sink strength in the tissues they attack (Arnold et al., 2004; Appel et al., 2012). Hexoses provide both signals and substrates for these and other defense responses (Schultz et al., 2013; Tauzin and Giardina, 2014). While we did not measure sink strength in these studies, the results support the view that hexose signaling and modified source-sink relationships are part of the plant-insect interaction.

Downregulation of protein synthesis is a common plant response to stress and is accomplished by phosphorylation of the alpha subunit of EUKARYOTIC INITIATION FACTOR 2 (eIF2alpha) by the kinase GENERAL CONTROL NON-REPRESSIBLE 2 (GCN2; Immanuel et al., 2012). Upregulation of GCN2 by *M. persicae* may represent a core regulatory response, but the downstream effects of eIF2alpha are not well enough understood in *Arabidopsis* to confirm its role in plant responses to insects (Li et al., 2013; Luna et al., 2014). Downregulation of protein translation is triggered by stress on the endoplasmic reticulum and likely involves the unfolded protein response (Duwi Fanata et al., 2013). This scenario may explain the increase in transcription of heat shock proteins observed in many transcriptome studies, including our own (Heidel-Fischer et al., 2014). However, downregulation of all protein synthesis following tissue damage is unlikely to be adaptive for the plant. Indeed, we observed in response to caterpillar feeding the upregulation of defense genes encoding proteins involved in biosynthesis and activity of glucosinolate biosynthesis, including their amino acid precursors. Similarly, the upregulation by aphids and downregulation by caterpillars of genes associated with cell wall metabolism are likely to reflect their differential impact on the TOR pathway controlling plant cell growth (Leiber et al., 2010; Henriques et al., 2014).

Fine tuning of metabolic programming to modulate investment in growth vs. defense has been reported previously (Schwachtje and Baldwin, 2008; Heidel-Fischer et al., 2014) and references therein. In *N. attenuata*, deactivation of ribulose bis-phosphate carboxylase (RuBPCase) by RUBPCAse activase mediates the change in primary metabolism which in turn attenuates JA-induced defense responses following chewing insect attack (Mitra and Baldwin, 2014). In *Arabidopsis*, DELLA proteins are also involved in attenuation of the JA response (Lan et al., 2014) but a role for RuBCase has not yet been examined.

### Transcriptional signatures of hormone signaling

It has become almost a truism to attribute plant responses to sucking insects as arising primarily from SA signaling and plant responses to chewing insects as arising primarily from signaling by JA and ethylene. However, the reality is much less clear-cut (Verhage et al., 2011; Erb et al., 2012) and signaling differences may in part be a matter of relative timing of herbivory along with importance of the herbivore (Appel et al., 2014; Rehrig et al., 2014) and a matter of ABA signaling. In this study, both chewing and sucking insects elicited the expression of genes associated with SA signaling, consistent with some reports of positive crosstalk between the two pathways (Schweiger et al., 2014) and the susceptibility of sucking insects to changes in JA signaling (Ellis et al., 2002; Mewis et al., 2005). However, the pattern was not reciprocal for JA; only caterpillars and mechanical wounding elicited expression of genes associated with JA signaling. Caterpillars also elicited the expression of genes associated with ethylene signaling, with evidence of differential induction of ethylene by the two species used in this study (Rehrig et al., 2014 *this volume*). Caterpillars uniquely elicited expression of genes associated with ABA signaling, which in *N. attenuata* is due in part to the inhibitory effect of an elicitor in caterpillar oral secretions on ABA catabolism (Dinh et al., 2013). Since ABA interacts with the SnRK1 sensors (Rodrigues et al., 2013), it is another likely source of hormonal crosstalk in plant responses to herbivores.

### *Cis*-elements of insect regulated genes explain only some of the transcriptional differences

Given evidence of insect-specific plant responses, we hypothesized that the *cis*-binding element composition of treatment-affected genes might display “fingerprints” characteristic of the treatment. No known regulatory elements have been found to be insect-specific in plants and little evidence is available about gene regulatory networks in plant-insect interactions (Zou et al., 2011). Some have proposed that a substantial part of the patterns in any gene expression data could be explained by “*cis*-element profiles” (Beer and Tavazoie, 2004; Segal et al., 2005). Using computational predictions with yeast stress data, some have argued that motif profiles in conjunction with microarray analysis can help identify important regulatory networks including those involved in the pathology of cancer development (Segal et al., 2005). There are only a few studies where this has been applied to plants. For example, using computational analysis and publically available *Arabidopsis* microarray databases, over 53 putative motifs involved in phytohormone signaling in plants were discovered (Yamamoto et al., 2011). The authors suggest using computational models as starting points for hypothesis formation before beginning wet-bench experiments. With this in mind, we characterized all of the *cis*-elements in promoter regions 1000 bp upstream of all genes affected by treatments using three different bioinformatic tools. Our results showed that the genes differentially expressed in any treatment differed in only a few known *cis*-elements in their promoter regions. However, a few elements were associated specifically with particular treatments and differed from randomly-selected TF gene profiles. First, *cis*-elements of TF genes down-regulated by *M. persicae* were quite different from any other set, consistent with the distinctive patterns of gene expression it elicited. Second, ABRE and ABRE-like elements were over-represented in most insect response treatments. This association is consistent with the role of ABREs in regulating the expression of genes that are ABA and drought-responsive (Fujita et al., 2005) and with the enrichment of ABA-responsive genes we observed in our caterpillar treatments. Although we did not detect a significant change in gene expression associated with ABA signaling in the aphid treatments of our study, others have reported them (Morkunas et al., 2011).

Third, TATA boxes were enriched in genes upregulated by both caterpillars; these elements may also be important regulatory components in biotic stress. Genes of *Saccharomyces sp*. with TATA boxes were found to be overrepresented in stress responses to abnormal osmolarity, pH balance, or nutrient availability (Basehoar et al., 2004). Genes without TATA boxes performed more constitutive housekeeping functions and may not need as much transcriptional regulation. The presence of TATA-binding motifs may identify *Arabidopsis* genes that are especially responsive to environmental stressors, especially chewing insects.

Taken together, these data suggest that several *cis*-elements distinguish the *Arabidopsis* gene regulatory networks involved in responses to insect attack, but that the promoter regions of responsive genes contain mostly the same elements. It is also likely that regulation of transcriptional responses to insects via TFs could be combinatorial, requiring multiple TFs to initiate particular expression patterns (Singh, 1998; Lindlöf et al., 2009; Zou et al., 2011).

In summary, our results provide a comprehensive overview of *Arabidopsis* transcriptional responses to chewing and sucking insects. We identified expression patterns in genes and *cis*-elements over-represented in co-expressed genes after insect attack that provide insight into the complex networks and regulatory pathways insects elicit in plants during herbivory. The results provide definitive evidence that plant responses to insects are distinct from and considerably more complex than are responses to wounding alone, even though insects wound as they feed. Thus, HAMPs are more important than DAMPS in determining plant responses to insects. Analyses of coexpression patterns and known interaction networks suggest that characterizing “master regulators” of plant responses to stress, including NPR1, Mediator Complex (Med25), and SnRK1 and TOR kinases is likely to provide additional insight (Robaglia et al., 2012; Balderas-Hernández et al., 2013; Sheen, 2014).

## Author contributions

Heidi M. Appel—Helped design array and insect experiments, conducted insect experiments, prepared manuscript, analyzed and interpreted data. Howard Fescemyer—Helped design microarray experiments, prepared RNA for microarray analysis, edited manuscript. Juergen Ehlting—Helped design and conduct microarray experiments. David Weston—Conducted network cluster analysis. Erin Rehrig—Conducted qRT-PCR validation of microarray and *cis*-element analysis, edited manuscript. Trupti Joshi—Provided advice on *cis*-element analysis, edited manuscript. Dong Xu —Provided advice on *cis*-element analysis, edited manuscript. Joerg Bohlmann—Helped design microarray experiments, edited manuscript. Jack Schultz—Helped design microarray and insect experiments, assisted with the analysis and interpretation of data, conducted hierarchical clustering, edited manuscript, authored funding for project.

### Conflict of interest statement

The authors declare that the research was conducted in the absence of any commercial or financial relationships that could be construed as a potential conflict of interest.
