# Supplementary material for: Transcriptional responses of Arabidopsis thaliana to chewing and sucking insect herbivores
Source: Front Plant Sci. 2014 Nov 14;5:565. doi: 10.3389/fpls.2014.00565 (PMC4231836; doi:10.3389/fpls.2014.00565)

**Supplemental Figure 1. Frequency distribution of parametric p-values from analysis of variance (ANOVA).** For each of the 26,090 probes on the microarray, normalized expression ratios from four replicate arrays for each of the four time points were used for ANOVA. The horizontal, blue line indicates the estimated NULL distribution separating the number of true positive tests (above the line) from negative tests within a given p-value bin (falsely discovered genes).


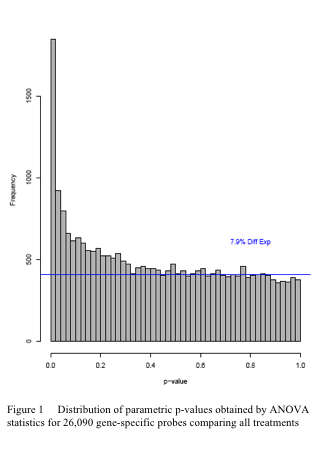
**Supplemental Figure 2. Differentially expressed genes associated with the ‘cell wall’ GO annotation** (0005618 for the cellular component ontology). Asterisks indicate genes in which the change in expression was statistically significant. Abbreviations for plant treatment names are the same as those used in Table 1.

**Supplemental Figure 3. Differentially expressed genes that are targets of SnRK1.** Asterisks indicate genes in which the change in gene expression was statistically significant. Abbreviations for plant treatment names are the same as those in Table 1.

**Supplemental Figure 4. Dendrogram (A) and architecture (B) of the weighted coexpressed gene network.** The red line in the dendrogram indicates the point above which modules were considered independent; modules below the line were merged with their neighbor modules. The non-random architecture reflects the importance of individual modules in shaping the gene network.

(A)

(B)**Supplemental Figure 5.** **The ATTED interaction network for WRKY 40** (yellow). Transcription factors are shown in hexagonal shapes; * = genes associated with the response to mechanical wounding, and + = genes associated with the response to ethylene.


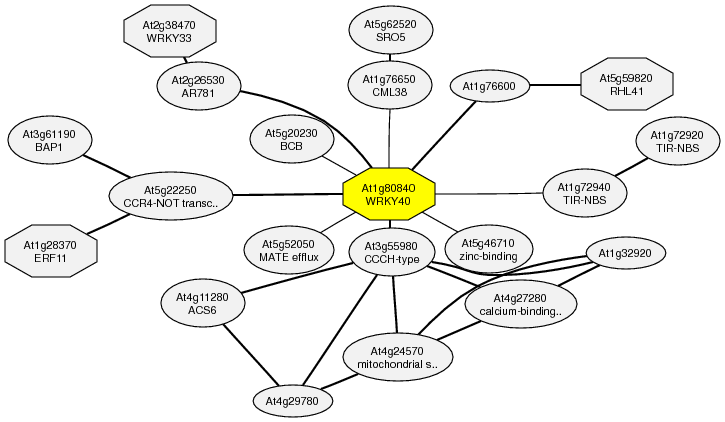

Supplement: Supplementary file 2 [file DataSheet1.DOCX]
